# Identification of Anti-virulence Compounds That Disrupt Quorum-Sensing Regulated Acute and Persistent Pathogenicity

**DOI:** 10.1371/journal.ppat.1004321

**Published:** 2014-08-21

**Authors:** Melissa Starkey, Francois Lepine, Damien Maura, Arunava Bandyopadhaya, Biljana Lesic, Jianxin He, Tomoe Kitao, Valeria Righi, Sylvain Milot, Aria Tzika, Laurence Rahme

**Affiliations:** 1 Department of Surgery, Harvard Medical School and Massachusetts General Hospital, Boston, Massachusetts, United States of America; 2 Department of Microbiology and Immunobiology, Harvard Medical School, Boston, Massachusetts, United States of America; 3 Shriners Hospitals for Children Boston, Boston, Massachusetts, United States of America; 4 INRS-Institut Armand Frappier, Laval, Québec, Canada; 5 NMR Surgical Laboratory, Department of Surgery, Massachusetts General and Shriners Hospitals, Harvard Medical School, Boston, Massachusetts, United States of America; 6 Athinoula A. Martinos Center of Biomedical Imaging, Department of Radiology, Massachusetts General Hospital, Boston, Massachusetts, United States of America; The University of Texas at Austin, United States of America

## Abstract

Etiological agents of acute, persistent, or relapsing clinical infections are often refractory to antibiotics due to multidrug resistance and/or antibiotic tolerance. *Pseudomonas aeruginosa* is an opportunistic Gram-negative bacterial pathogen that causes recalcitrant and severe acute chronic and persistent human infections. Here, we target the MvfR-regulated *P. aeruginosa* quorum sensing (QS) virulence pathway to isolate robust molecules that specifically inhibit infection without affecting bacterial growth or viability to mitigate selective resistance. Using a whole-cell high-throughput screen (HTS) and structure-activity relationship (SAR) analysis, we identify compounds that block the synthesis of both pro-persistence and pro-acute MvfR-dependent signaling molecules. These compounds, which share a benzamide-benzimidazole backbone and are unrelated to previous MvfR-regulon inhibitors, bind the global virulence QS transcriptional regulator, MvfR (PqsR); inhibit the MvfR regulon in multi-drug resistant isolates; are active against *P. aeruginosa* acute and persistent murine infections; and do not perturb bacterial growth. In addition, they are the first compounds identified to reduce the formation of antibiotic-tolerant persister cells. As such, these molecules provide for the development of next-generation clinical therapeutics to more effectively treat refractory and deleterious bacterial-human infections.

## Author Summary

Antibiotic resistant and tolerant bacterial pathogens are responsible for acute, chronic and persistent human infections recalcitrant to any current treatments. Therefore, there is an urgent need to identify new antimicrobial drugs that will help circumvent the current antibiotic resistance crisis. Bacterial pathogens often develop resistance to antibiotic drugs that target bacterial growth or viability. In contrast, strategies that specifically target virulence pathways non-essential for growth could limit selective resistance, and thus are candidates for the development of next-generation antimicrobial therapeutics. In this study we target the bacterial communication system MvfR (PqsR), which is known to control virulence of the opportunistic bacterial pathogen *Pseudomonas aeruginosa*. We identified and improved upon new small molecules that effectively silence the MvfR communication system, and as a result block *P. aeruginosa* virulence both *in vitro* and *in vivo*. Moreover, these new compounds are the first known to restrict the ability of bacteria to form antibiotic-tolerant cells and consequently proved to be very effective at preventing persistent infection in a mammalian infection model. Because of their ability to simultaneously block acute and persistent infections, these new molecules may provide a very strong basis for the development of next generation antimicrobials.

## Introduction

Antibiotic-resistant and tolerant microbes mediate acute, persistent, chronic, and/or relapsing human infections [1]–[3]. Such infections occur worldwide, affect all sectors, cause physical and emotional suffering, impose high financial costs on patients and healthcare systems, and are refractory to current anti-infective drugs. As such, identification of new molecular targets and corresponding compounds to restrict multidrug-resistant (MDR) and antibiotic-tolerant (AT) infections will substantively benefit human healthcare.

Bacterial pathogens often develop resistance to antibiotic drugs that target bacterial growth or viability. In contrast, strategies that specifically target virulence pathways that are non-essential for growth could limit selective resistance, and thus are candidates for the development of next-generation antimicrobial therapeutics. One candidate pathway is quorum sensing (QS), a cell-to-cell density-dependent communication system mediated via the production of and regulation by low molecular weight signaling molecules. QS, which is evolutionarily conserved throughout eubacteria and archaebacteria, is crucial for the development and maintenance of acute and chronic/persistent human infections as well as the commonly observed antibiotic tolerance of many pathogenic bacteria [4]–[10]. As such, anti-virulence compounds that specifically target QS could have a major impact on the control and treatment of a wide-range of acute and persistent bacterial infections [11]–[13].

*P. aeruginosa* is a wide-spread opportunistic human pathogen responsible for acute and chronic/persistent infections that readily develop multi-drug resistance to clinical antibiotics, and often evade clinical treatment [1]–[3]. *P. aeruginosa* has three distinct QS systems mediated by cell-to-cell signals including the acyl-homoserine lactones (HSL) 3-oxo-C12-HSL and C4-HSL, respectively produced by the las and rhl QS systems; and the 4-hydroxy-2-alkylquinolines (HAQs), produced by the mvfR (pqsR) QS system [14]. MvfR is a LysR-type transcriptional regulator (LTTR) that directs the synthesis of ∼60 low molecular weight HAQ molecules, including its positive regulatory ligands 4-hydroxy-2-heptylquinoline (HHQ) and 3,4-dihydroxy-2-heptylquinoline (PQS); and the non-HAQ, 2-aminoacetophenone (2-AA) [7], [15]–[16]. LTTRs control the expression of a diverse array of virulence regulons in Gram-negative and Gram-positive pathogens, and are the largest family of homologous regulators in prokaryotes [17].

While all three *P. aeruginosa* QS systems are required for full pathogenicity in mammalian hosts [18]–[21], the lasR pathway is often inactivated in isolates from cystic fibrosis (CF) patients, and thus it may be nonessential for chronic/persistent infections. This inactivation is due to mutations in LasR itself [22], [23], and may be due to specific MvfR-regulated functions [7]. Conversely, MvfR is essential for full virulence in several host models [19], [24], [25], and clinical isolates with *mvfR* mutations have not been identified. MvfR binds to and activates the *pqs* operon, which encodes enzymes for the synthesis of HAQs, including PQS and HHQ [15], [16], [26]; and for MvfR-regulated small molecules, including 2-AA. These molecules are produced in human tissues and function in pathogenicity [27], [28]. Both HHQ and PQS bind to and activate MvfR [16], [26] to lead to the production of MvfR-regulated virulence factors that promote acute infections [25], [29]–[31]. 2-AA, which is produced in human tissues [32], signals changes in both bacterial [7] and host pathways [6], [33]. Some of the affected pathways underlie the development and maintenance of chronic/persistent infections, including functions that promote antibiotic tolerance [8], long-term survival and persistence [7], and modulation of host functions that promote pathogen tolerance [6].

Antibiotic-tolerant (AT) cells underlie bacterial persistence and correspond to sub-populations that survive lethal concentrations of antibiotics. AT cells are implicated in the clinical failure of antibiotic therapy, and may populate and/or be responsible for persistent infections that can be the source of latent, chronic, or relapsing infections that are suppressed but not eradicated by antibiotics [34]–[36].

MvfR, due to its central role in both acute and chronic/persistent infections, is a potential target for the development of new anti-microbial drugs, especially as it is nonessential for cell viability or growth. Here we identify robust quorum sensing inhibitors (QSI) that inhibit the MvfR virulence regulon via binding to the MvfR regulatory protein; are highly efficacious in disrupting MvfR-dependent cell-to-cell communication *in vivo*; and limit *P.* aeruginosa infections and lethality in mice. Moreover, these are the first identified compounds that restrict the formation of antibiotic-tolerant persister cells, and consequently, that restrict *P. aeruginosa* persistent infections in mice. These molecules, which belong to a chemical family previously unrecognized for MvfR inhibitory activity, provide for the development of effective clinical therapeutics to limit and eradicate acute and chronic/persistent multi-drug resistant infections.

## Results

### High-throughput whole-cell screening identifies novel potent MvfR-regulon inhibitors with a benzamide-benzimidazole chemical backbone

**Figure 1:**
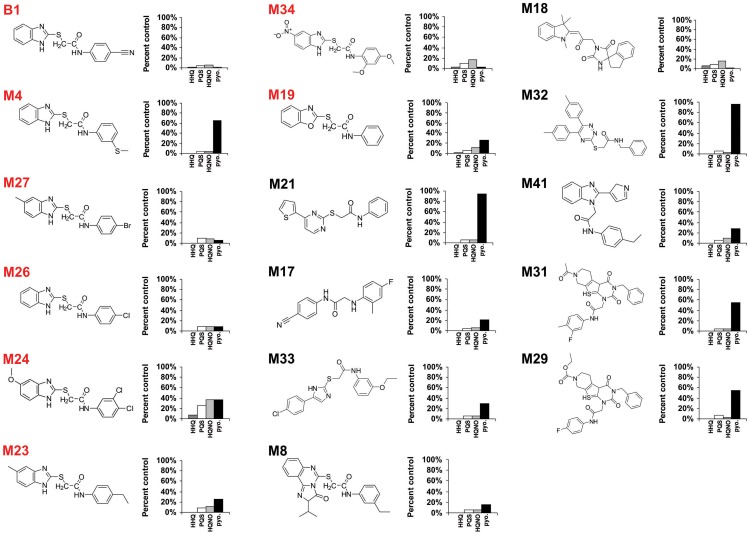
Chemical structures of 17 MvfR-regulon inhibitors identified by whole cell HTS, and their corresponding inhibition of HAQ and pyocyanin production. HHQ (dark grey bars), PQS (white bars), HQNO (light grey bars), and pyocyanin (black bars) levels were quantified plus or minus 50 µg/mL of each compound. Structures labelled in red share the common benzamide-benzimidazole core.

We used a whole cell high-throughput screen (HTS) to identify compounds that inhibit MvfR regulon activity without perturbing cell viability or growth (Fig. 1, S1 and S2b). We screened a chemical library of 284,256 low molecular weight compounds for inhibition of *pqsA* expression using a reporter consisting of the *pqsA* promoter fused to the *sacB* gene (Fig. S1) [7], [37]. In this screen, a solvent control or non-inhibitory compound results in bacterial death when sucrose is present in the culture media due to its conversion to toxic levans by the *sacB* gene product [37], while compounds that inhibit *pqsA* promoter expression, and thus HAQ synthesis, permit bacterial growth.

**Figure 2:**
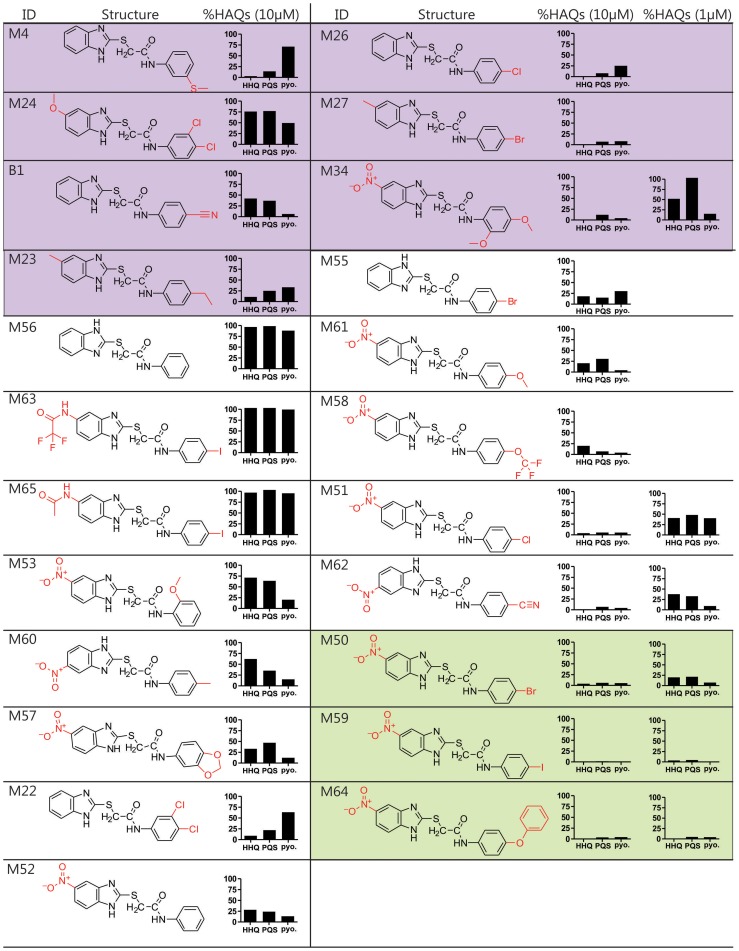
Structure and biological activity of benzamide-benzimidazole derivatives for inhibition of HAQ and pyocyanin production. The HTS compounds are shaded purple, the 2^nd^ generation commercially available derivatives are shaded white, and the 2^nd^ generation synthetic derivatives are shaded green. Alterations to the M56 benzamide-benzamidazole core structure are marked in red. HHQ, PQS, and pyocyanin (pyo.) levels were quantified in response to 10 µM compounds, and 1 µM of the most potent compounds: M34, M51, M62, M50, M59, and M64.

The MvfR-regulon inhibitory compounds initially identified belong to 7 distinct chemical families (Fig. 1). The most effective inhibitors were verified via a second screen for *pqsA* promoter repression using a *pqsA-*GFP [38] reporter construct, which yielded 39 candidate compounds (Figs. S1 and S2a). These inhibitors were further analyzed by functional assays for reduced HAQ production, including HQNO, and the MvfR ligands HHQ and PQS; and for reduced levels of pyocyanin, an MvfR-regulated virulence effector. Figure 1 presents the structures and LC/MS results for 17 compounds that completely eliminated *pqsA*-GFP mediated fluorescence, greatly reduced HHQ, PQS, and HQNO levels at 50 µg/mL, and notably, did not impact bacterial growth (Fig. S2). Some of these compounds also eliminated or greatly reduced pyocyanin levels (Fig. 1). Strikingly, 8 of these compounds (labeled in red) share a benzamide-benzimidazole (BB) backbone, consisting of a substituted benzamide moiety and endocyclic aromatic amines. These BB inhibitors also increased anthranillic acid (AA) in culture (*data not shown*), likely via its non-utilization and subsequent accumulation [15], [39]. The other 9 inhibitors presented in Figure 1 are unrelated to the BB inhibitors and do not share any common structure or distinct feature between them. Also, 12 of the total 17 inhibitors reduced pyocyanin to less than 50%, while the BB compound M4 and the non-BB compounds M21, M29, M31, and M32, did not (Fig. 1). All BB inhibitors identified from the HTS, except for M24, reduced HHQ and PQS levels to ≤15%, and M4, M23, M26, M27, and M34 were effective at ≤10 µM (Fig. 2). This concentration is 150 fold lower than the effective concentrations for previously identified MvfR-regulon inhibitors [40].

### Structure-activity relationship analysis identifies improved 2^nd^ generation benzamide-benzamidazole MvfR-regulon inhibitors

We performed a structure-activity relationship (SAR) analysis to define 2^nd^ generation BB derivatives with enhanced MvfR-regulon inhibitory activity, as determined by repression of HAQ and pyocyanin production at 10 µM and 1 µM (Fig. 2). Starting with M56, increased inhibitor activity was obtained by adding an electron withdrawing group, such as a nitro, to position 5 of the benzimidazole moiety, to give M52; or an electron-releasing group to the benzamide *para* position, to give M55. As such, we focused on compounds with a nitro substituted benzimidazole ring and a *para* substituted benzamide ring. Derivatives having a methoxy (M61), trifluoromethoxy (M58), chloro (M51), or a cyano (M62) *para* group showed increased inhibitory activity at ≤1 µM IC_50_ (Fig. 2, white). For further optimization, we synthesized BB derivatives containing a nitro substituted benzimidazole ring and a *para*-bromo (M50), iodo (M59), or phenoxy (M64) substituted benzamide ring (Fig. 2, green). Also, the BB thioether bond was critical for inhibition in M59, as replacing it with a methylene eliminates activity (*data not shown*). M64 was the most effective 2^nd^ generation inhibitor for reducing PQS, HHQ, and pyocyanin production, with respective IC_50_ of 200 nM, 350 nM, and 300 nM (Fig. S3).

### MvfR regulon inhibitors restrict the formation of *P. aeruginosa* antibiotic tolerant cells

**Figure 3:**
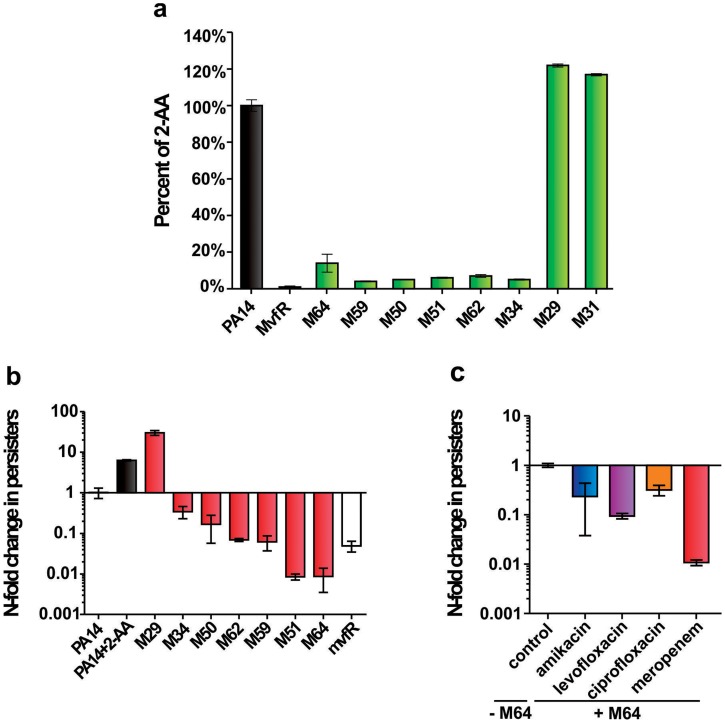
The most potent inhibitors reduce 2-AA production and the formation of antibiotic tolerant persisters. **a.** 2-AA levels in presence of 10 µM inhibitor. Error bars show mean +/− SD of at least 2 replicates. **b.** Observed fold change in persister cell concentrations of PA14 cultures with 10 µM inhibitor or with 0.75 mM 2-AA. Untreated PA14 cells and *mvfR-* cells were the positive and negative controls, respectively. Error bars show mean +/− SEM of at least 3 replicates. Differences between PA14 and the samples M34, M50, M62, M59, M51, M64 or mvfR- (*p*<0.01) as well as between PA14 and the samples PA14 + 2-AA or M29 (*p*<0.01) are statistically significant (one way ANOVA, Dunnett's test). **c.** Observed fold change in persister cell concentrations of PA14 plus 5 µM M64 in the presence of clinical antibiotics used to treat *P. aeruginosa* infections: amikacin (blue), levofloxacin (purple), ciprofloxacin (orange) and meropenem (red). All values were normalized to control cultures in 0.01% DMSO. Error bars show mean +/− SEM of at least 3 replicates. Differences between control and the samples amikacin, levofloxacin, ciprofloxacin or meropenem are statistically significant (*p*<0.01, one way ANOVA, Dunnett's test).

2-AA, an abundant MvfR-regulated non-HAQ small molecule, promotes *P. aeruginosa* antibiotic-tolerant (AT) cell formation [8], [41] and bacterial persistence in infected flies [7] and mice [6]. Figure 3 shows that the MvfR BB inhibitors prevented both 2-AA synthesis and AT cell accumulation, suggesting their potential to limit *P. aeruginosa* chronic/persistent infections.

Figure 3a shows that the 6 most potent BB inhibitors of HAQ and pyocyanin production (M34, M50, M51, M59, M62 and M64) dramatically reduced 2-AA production; while two potent HAQ non-BB inhibitors, M29 and M31, conversely, slightly increased 2-AA production (Fig. 3a). Figure 3b shows that all of the BB compounds that decreased 2-AA production also decreased the number of persister cells tolerant to the β-lactam antibiotic meropenem while alternatively, M29 increased persisters, perhaps via increased 2-AA.

**Figure 4:**
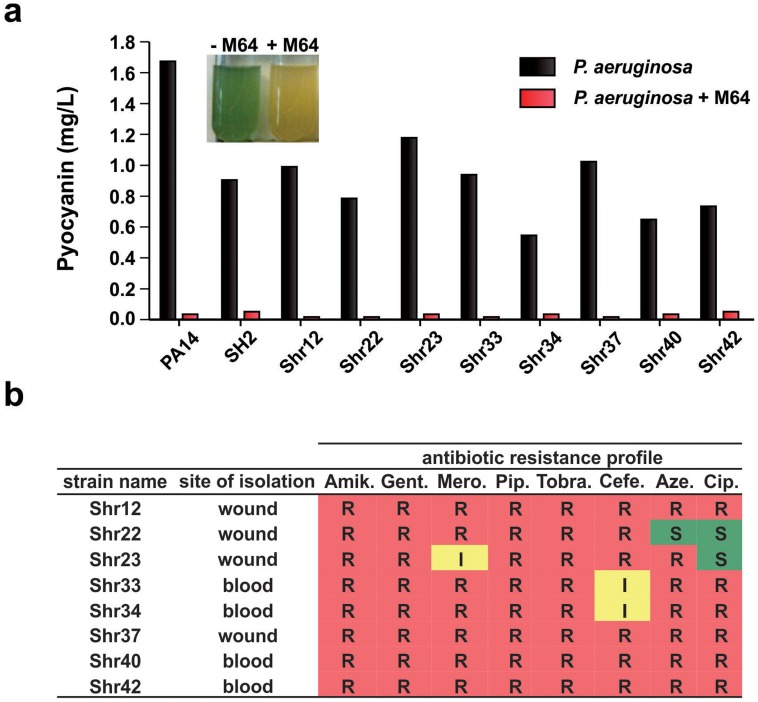
M64 reduces pyocyanin production in *P. aeruginosa* clinical multi-drug resistant strains. **a.** Quantitative pyocyanin production in multi-drug resistant clinical *P. aeruginosa* isolates plus (red) and minus (black) 5 µM M64. A representative image of qualitative pyocyanin production, visible as green media, in PA14 culture +/− M64, is shown above the histogram. **b.** Antibiotic resistance profile of *P. aeruginosa* clinical strains and their respective isolation sites from infected patients. Amik. = amikacin, Gent. = gentamycin, Mero. = meropenem, Pip. = piperacin, Tobra. = tobramycin, Cefe. = cefepime, Aze. = azetromycin, Cip. = ciprofloxacin. R = resistant; I = intermediate; S = sensitive.

We focused on the BB compounds that restrict persister formation. Figure 3c shows the generalized anti-persister efficacy of M64, as it limits formation of antibiotic-tolerant persisters to other antibiotic classes, including quinolones (ciprofloxacin, and levofloxacin), and aminoglycosides (amikacin). In addition, M64 inhibited pyocyanin production in several *P. aeruginosa* clinical isolates (Fig. 4a), including multidrug or pan-resistant strains (Fig. 4b), suggesting its potential for the development of anti-infective reagents against recalcitrant MDR strains.

### Mechanism of MvfR regulon inhibition

**Figure 5:**
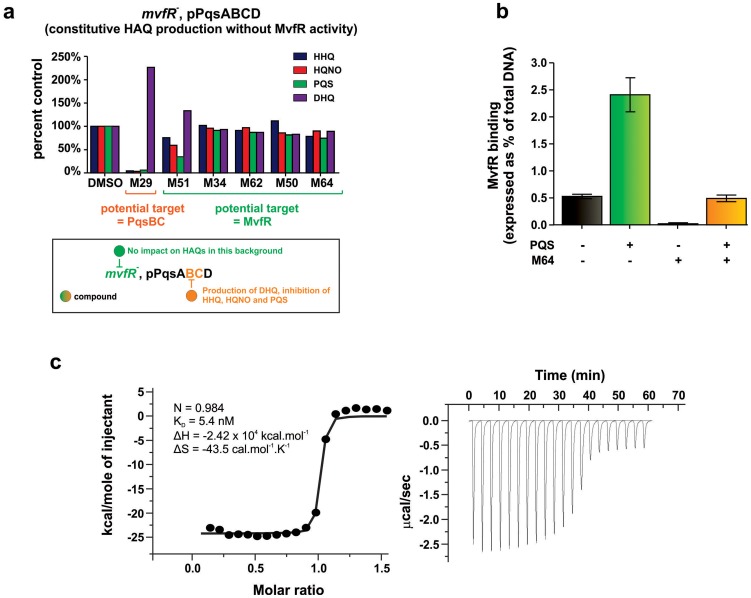
Identification of the molecular target and mode of action of the MvfR-regulon inhibitors. **a.** The potential molecular target of an inhibitor is revealed by the pattern of HAQ and DHQ production in response to 100 µM of the inhibitor in *mvfR* mutant cells that constitutively express *pqsABCD*. Compounds that target MvfR should not impact HAQs production (e.g., M51, M34, M62, M50, M64); compounds that inhibit the PqsB or PqsC enzymes should result in increased DHQ, which is produced by PqsA/D, and reduced levels of HHQ, PQS, and HQNO, which require the PqsABCD enzymes (e.g., M29). **b.** 0.24 µM M64 blocks MvfR binding to the *pqsA* promoter of PA14, minus and plus 38 µM PQS. Binding was assessed by ChIP-qPCR and normalized to the *rpoD* promoter that lacks an MvfR binding site. MvfR bound DNA was expressed as the percent of total input DNA. Error bars represent mean +/− SEM of at least 3 replicates. **c.** Isothermal titration calorimetry analysis of the interaction between MvfRc91 (19 µM) and M64 (200 µM). Heat signals of the M64 titration into MvfRc91 are plotted against the molecular ratio between M64 and MvfRc91 (left panel), and against time (right panel). The best-fit curve corresponds to a single-site binding model. The stoichiometry of binding (N), association constant (K_A_), enthalpy (ΔH), and entropy of binding (ΔS) are presented.

The mechanism of action of the most potent MvfR inhibitors is not obvious, as their common BB backbone is unrelated to MvfR ligands or the biosynthetic precursors or intermediates of these ligands. As these compounds decrease HAQ and 2-AA production, they might target the MvfR regulatory protein, or alternatively, the *pqs* operon enzymes that mediate HAQ and 2-AA biosynthesis [15]. To this end, we asked if the MvfR-regulon inhibitors reduce HAQs, as assessed by LC/MS, in an isogenic *mvfR* mutant strain that constitutively expresses the *pqsABCD* genes, and thus has MvfR-independent HAQ production. Figure 5a shows that the most potent BB inhibitors (M51, M34, M62, M50 and M64) did not alter HAQ levels compared to the solvent control, suggesting that they target MvfR or another upstream regulatory component. In contrast, the non-BB inhibitor, M29, reduced HHQ, PQS, and HQNO levels, and increased DHQ (Fig. 5a). Since PqsA and PqsD are necessary and sufficient for DHQ production [42], [43], M29 may inhibit the PqsB and/or PqsC enzymes that are required for HHQ and PQS, but not DHQ or 2-AA production [7].

An essential step of *pqsA* promoter activation is the binding of the MvfR protein to specific DNA residues within the *pqs* promoter, which is enhanced by PQS or HHQ [16]. To determine whether M64 disrupts this binding, PA14 cells expressing MvfR fused to a vesicular stomatitis virus glycoprotein (VSV-G) epitope at the C-terminus were grown with and without M64, and the MvfR–DNA complex was isolated via chromatin immunoprecipitation (ChIP). The co-precipitated DNA was quantified by qPCR [19], [44]. Figure 5b shows that M64 decreased MvfR binding to the *pqsA* promoter by ∼10-fold and blocked the PQS – mediated increase in MvfR binding (Fig. 5b). As shown in Figure S5, PQS and/or M64 addition did not affect MvfR levels.

Figure 5 demonstrates that the M64 molecular target is MvfR itself, rather than an upstream component. Isothermal titration calorimetry (ITC) showed that this binding has a ∼1∶1 stoichiometry for M64 and the MvfR co-inducer binding domain, with a K_D_ = 5.4 nM (Fig. 5c). This binding likely prevents MvfR binding to the *pqsA* promoter to inhibit MvfR regulon activation.

### MvfR regulon inhibitors attenuate *P. aeruginosa* acute virulence

**Figure 6:**
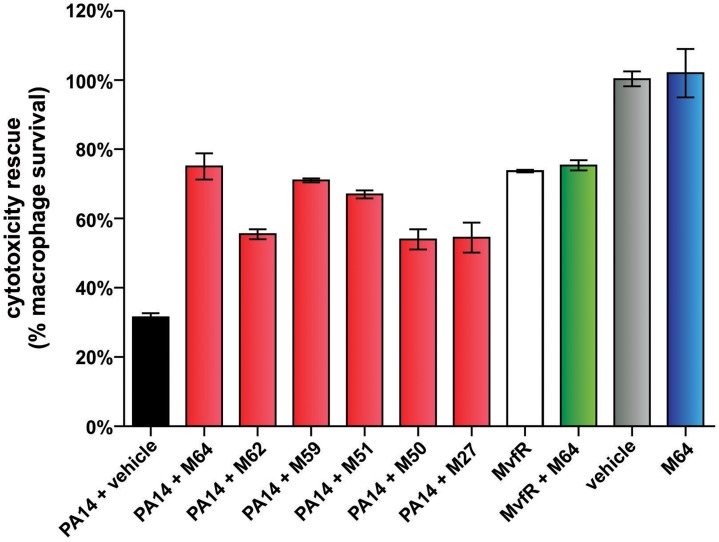
MvfR-regulon inhibitors rescue PA14-macrophage cytotoxicity. PA14-induced killing of Raw264.7 macrophage cells was determined minus and plus 100 µM inhibitor. Error bars represent mean +/− SEM of at least 3 replicates. Differences between PA14 + vehicle and the samples PA14 + M64, PA14 + M62, PA14 + M59, PA14 + M51, PA14 + M50, or PA14 + M27 are statistically significant (*p*<0.01, one way ANOVA, Dunnett's test). Differences between MvfR and MvfR + M64 (*p*>0.05) or vehicle and M64 (*p*>0.05) are not statistically significant (unpaired t test). Notably, M64 does not alter cytotoxicity of *mvfR* cells, and is itself non-cytotoxic.

**Figure 7:**
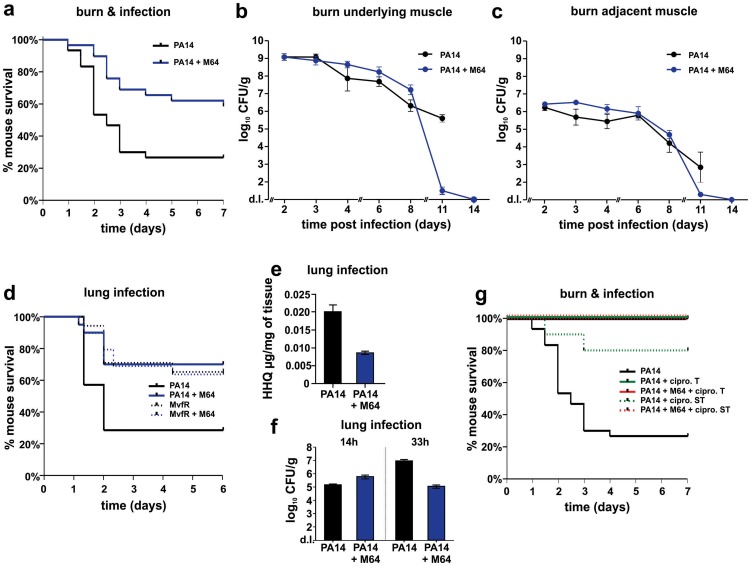
M64 reduces PA14 virulence in mouse burn infection, and lung infection, models. **a.** Survival curves of mice from the burn and infection model following PA14 infection, minus (black, n = 30), and plus (blue, n = 36), M64 (4 mg/kg). M64 was administered by intravenous injection 6 h post-burn and infection, and then twice a day for 6 days post-infection. Differences between PA14 and PA14 + M64 are statistically significant (*p*<0.001, Kaplan-Meier method). **b., c.** M64 does not significantly reduce PA14 bacterial load within the first 7 days post-infection, and alternatively promotes bacterial clearance over several days in the burn and infection model. PA14 CFU quantification in muscle underlying (b) or adjacent to (c) the abdominal infection site in mice infected with PA14, minus (black) and plus (blue) M64. Error bars show mean +/− SEM of at least 3 replicates. Animals that survived infection post-day 7 were used for CFU quantification at days 8 and 11. Differences between PA14 and PA14 + M64 are statistically significant at day 11 (*p*<0.001, unpaired t test) but not before day 11 (*p*>0.05, unpaired t test). **d.** Survival curves of mice from the lung infection model following PA14 infection, minus (solid black line, n≥10), and plus (solid blue line, n≥10) M64 (4 mg/kg); and *mvfR* infection, minus (interrupted black line, n≥10), and plus (interrupted blue line, n≥10) M64 (12 mg/kg). M64 was administered by intravenous injection at 2, 4, 8, and 12 h post infection, and then twice a day up to day 4. Differences between PA14 and PA14 + M64 (*p*<0.05) or between mvfR and mvfR + M64 (*p*<0.05) are statistically significant, while differences between mvfR and mvfR + M64 (*p*>0.05) or between PA14 + M64 and mvfR + M64 (*p*>0.05) are not statistically significant (Kaplan-Meier method). Animals were inoculated intranasally with 20 µL of 5×10^6^ PA14 cells and 20 µl of 8×10^6^ isogenic *mvfR* mutant cells, n≥10 mice. **e.** HHQ levels at 14 h post-infection from lung tissues in untreated mice, and from mice treated with M64. n = 7 for each experimental condition. Difference between PA14 and PA14 + M64 is statistically significant (*p*<0.001, unpaired t test). **f.** PA14 pulmonary bacterial load in mice infected with PA14 (black) and treated with M64 (blue) quantified at 14 or 33 h post infection. Error bars represent mean +/− SEM of at least 3 replicates. Difference between PA14 and PA14 + M64 at 33 h are statistically significant (*p*<0.05, unpaired t test) whereas difference at 14 h was not (*p*>0.05, unpaired t test). d.l., detection limit. NS, not significant. **g.** Survival curve of PA14-infected mice from the burn and infection models, untreated (black, n = 30), treated with ciprofloxacin (green, *p<0.001*), or treated with a combination of ciprofloxacin and M64 (red, *p<0.001*), using a 10 mg/kg therapeutic dose (T, straight line, n = 18–24, *p<0.001*) or a 4 mg/kg sub-therapeutic dose (ST, dashed line, n = 10, p<0.001) of ciprofloxacin. Ciprofloxacin was administered by intravenous (IV) injection twice a day for 4 days post-infection, and M64 was administered by IV injection 6 hours post-infection and then twice a day for 6 days post-infection. In all conditions the M64 dose was 4 mg/kg. Significance of survival rate differences compared to PA14 infected mice was determined using the Kaplan-Meier method.

**Figure 8:**
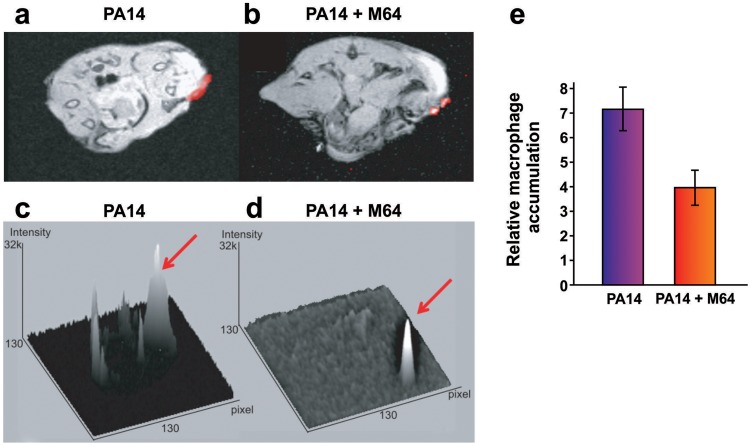
Magnetic resonance imaging of M64 inhibition of macrophage recruitment at a burn and infection site. **a–e.** *In vivo* positive contrast imaging of mice infected with PA14, plus and minus M64. The off resonance imaging transverse relaxation in the rotating frame (ORI-T2ρ) images were transformed to signal to noise ratio (SNR) images and thresholded in units of image standard deviation. **a., b.** The positive-contrast images are presented in pseudocolor, thresholded to signal greater than three in dimensionless SNR units, and superimposed on a FLASH image. For image processing, regions of interest (ROI) were drawn around the burn region and the total thresholded signal intensity was integrated within each ROI. Similar slices were chosen at the same anatomical location in all mice. **c., d.** 3-dimensional graphs of pixel intensities show an intense peak in the burn area for the PA14 control mouse, with this peak reduced by M64. **e.** Signal was measured in units of SNR, thresholded at three standard deviations, and measured within ROIs at the level of the burn and infection. The noise threshold was estimated by fitting the image background to a Rician distribution. Error bars depict standard error of the mean image intensity in the ROI. Error bars depict mean +/− SD of at least 3 replicates. Difference between PA14 and PA14 + M64 is statistically significant (*p*<0.05, unpaired t test).

**Figure 9:**
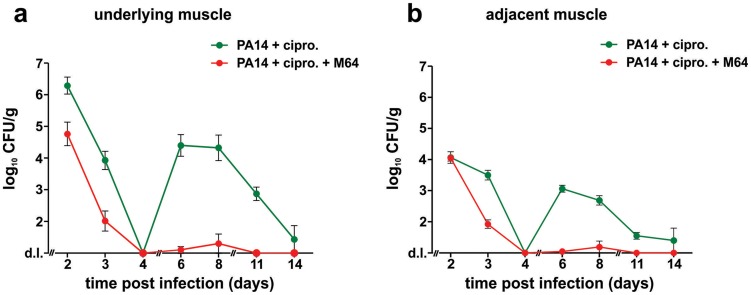
M64 inhibits *P. aeruginosa* persistence in the mouse burn and infection models. PA14 CFU quantification in muscle (**a**) underlying or (**b**) adjacent to the abdominal infection site in mice infected with PA14 and treated with ciprofloxacin (10 mg/kg), and minus (green) or plus (red) M64 (4 mg/kg). Ciprofloxacin and M64 were administered by intravenous injection 6 hours post-infection and then twice a day. Ciprofloxacin was administered for 4 days as described until no CFUs were detected in the muscle samples. Ciprofloxacin administration was stopped at day 4 to allow for the potential emergence and detection of antibiotic-tolerant cells. M64 was administered for 6 days, up until antibiotic-tolerant cells were detected in the PA14 + ciprofloxacin only group. Error bars represent mean +/− SEM of at least 3 replicates. d.l., detection limit.

MvfR QS is a target pathway for the development of new anti-infective reagents, as it controls a large regulon of virulence functions [16], [25] and is required for pathogenicity in evolutionary distinct hosts [19], [20], [24], [31], [45]–[47]. To this end, we asked if our MvfR inhibitors limit bacterial virulence in murine macrophages (Fig. 6) and in mice (Figs. 7–9). Figure 6 shows that the BB inhibitors significantly reduced PA14 cytotoxicity in Raw 264.7 macrophages. M64 was the most effective compound and was not cytotoxic to the macrophages. 76% of PA14-infected cells survived in the presence of M64 compared to only 36% survival in the absence of this compound *(p<0.01)*. In addition, *mvfR* mutant cells were less cytotoxic than wild-type PA14, and M64 did not rescue this cytoxicity, further confirming that it targets the MvfR pathway.

Two distinct *in vivo* murine models of acute infection (thermal injury and lung infection) were used to ask if M64 is therapeutically efficacious for *P. aeruginosa-*mouse pathogenesis [40], particularly for highly virulent strains exemplified by PA14. Burned and infected mice that were injected with M64 beginning 6 h post-infection followed by injections twice daily through day 6, exhibited increased survival compared to control mice (*p<0.001*), demonstrating M64's anti-infective efficacy in mice to reduce PA14 acute virulence (Fig. 7a). This increased host survival was not due to reduced bacterial loads, as both treated and untreated animals had similar CFUs at the infection site or at the adjacent muscle up to 6 days post-infection (Fig. 7b and 7c). As such, M64 directly reduced acute PA14 virulence in mice, likely by inhibiting virulence functions, as opposed to reducing bacterial loads. Using this model we compared the M64 sensitivity of colonies isolated from infected versus infected and M64 treated animals. M64 sensitivity was assessed at day 11 post-infection and treatment by measuring M64 IC_50_ for pyocyanin production in 10 remaining colonies per animal (n = 3 animals per group), reasoning that mutants resistant to M64 would still produce pyocyanin in the presence of M64. No difference in M64 IC_50_ was observed in any of the tested colonies, suggesting that resistance does not arise at least by day 10 post-treatment (data not shown).

The *in vivo* anti-infective activity of M64 was further confirmed in a murine acute lung infection model (Fig. 7d), as M64 significantly reduced the mortality of PA14 infected mice by ∼2.5-fold (*p<0.05*), comparable to that of animals infected with attenuated *mvfR* mutant bacteria. M64 did not further rescue the reduced mortality of *mvfR*-infected mice (Fig. 7d). In addition, *in vivo* HHQ levels were significantly decreased in infected animals treated with M64 (*p<0.01*) (Fig. 7e) and the pulmonary bacterial loads were unchanged in treated versus untreated animals at 14 h post-infection. These results suggest that the *in vivo* specificity of M64 to inhibit MvfR regulon function reduces virulence while not affecting bacterial viability. Nonetheless, Figure 7f shows that bacterial loads in the lungs were significantly reduced in the treated animals by 33 h post-infection, suggesting that M64 treatment facilitated host clearance. A similar effect is also observed in the muscle samples from burned and infected mice treated with M64 (Fig. 7b and 7c).

M64 combined with a sub-therapeutic (ST) dose of ciprofloxacin in the thermal injury and infection model resulted in 100% survival of PA14-infected mice versus 80% survival for mice receiving ST ciprofloxacin alone *(p<0.001)*, suggesting an additive effect of M64 and ciprofloxacin (Fig. 7g). This effect is not due to altered ciprofloxacin minimal inhibitory concentration (MIC) (Table S1), and instead may be the consequence of differences in the mode of actions of these two compounds. Indeed, as the ciprofloxacin ST dose did not kill all of the PA14 cells, M64 might have reduced the virulence of the remaining cells to be cleared by the host immune system. Despite treatment ending at day 6, M64 monotherapy led to a dramatic decrease in bacterial CFUs in both muscle samples of burned and infected mice post-day 8, with complete bacterial tissue clearance by day 14 (Fig. 7b and 7c), demonstrating efficacy of M64 without the combinatorial antibiotic. None of the infected but untreated animals survived past day 11; hence no further samples could be taken from this population.

### M64 attenuates macrophage accumulation at the infection site

We used a Magnetic Resonance Imaging (MRI) technique to evaluate M64 anti-infective efficacy in live animals by monitoring accumulation of macrophages, which are a marker of host anti-pathogen functions at active infection sites. We introduced a novel combination of off resonance imaging (ORI) positive-contrast MRI and T2ρ relaxation in the rotating frame (ORI-T2ρ) for positive-contrast MR imaging of ultra-small superparamagnetic iron-oxide (USPIO) nanoparticles. This technique exploits the chemical shift induced by USPIO nanoparticles engulfed by macrophages to nearby water molecules. Macrophages accumulate at the *P. aeruginosa* infection site in response to bacterial virulence factors and host immune response functions [48]. Figure 8 demonstrates, via *in vivo* MRI, that M64 reduced macrophage accumulation at the *P. aeruginosa* infection site in the murine burn and infection model, corroborating its anti-infective potential. M64 is not cytotoxic to murine macrophages in culture, supporting the notion that the reduced macrophage accumulation is via inhibition of PA14-mediated inflammation rather than macrophage killing.

### M64 inhibits *P. aeruginosa* persistence in infected mice

Bacterial AT cells are likely a key component of latent, chronic, persistent, and relapsing infections, as they are a reservoir for re-initiation of active infections, and have been implicated in antibiotic treatment failures and host mortality [36], [49], [50]. To this end, new clinical therapies are needed that limit the ongoing presence of AT cells in infected hosts. Our inhibitors prevent 2-AA synthesis, which promotes the formation of *P. aeruginosa* persister cells [8], and persistence *in vivo* [6], [7].

We developed a mouse infection model to assess *P. aeruginosa* persistence *in vivo* following antibiotic treatment, and to evaluate M64 *in vivo* efficacy to reduce PA14 persistence in infected animal tissues, plus and minus combinatorial antibiotic therapy. Using this model, we demonstrate in Figure 9 that M64 inhibits persistence in the infected host, suggesting its broad anti-infective potential. No bacterial CFUs were detected in muscle tissue directly below or adjacent to the host infection site 4 days post-infection in animals treated with ciprofloxacin and M64, or with ciprofloxacin alone. Conversely, CFUs reappeared 6 days post-infection in the antibiotic-only treated animals, but not in those receiving antibiotic plus inhibitor. The co-injected animals exhibited minimal to no CFUs 6–8 days post-infection, with complete clearance by day 11 in the rectus abdominus muscle directly below or adjacent to the infection site, while the CFUs in the antibiotic-only animals were ∼10^3^–10^4^ 6–8 days post-infection and ∼100 cells by day 14 (Fig. 9a and 9b). These CFUs were confirmed to be antibiotic sensitive, as cultures inoculated from single surviving colonies from animals at days 6, 8, and 11 had identical ciprofloxacin MICs and killing curves compared to the parental PA14 culture (*data not shown*).

These data demonstrate the additive effect of M64 on ciprofloxacin to fully clear infection and indicate that M64 prevents tissue re-colonization after antibiotic treatment is stopped. This effect is not due to altered ciprofloxacin MIC (Table S1), further substantiating the anti-persistence efficacy of the MvfR regulon inhibitors.

## Discussion

The ideal anti-infective to treat bacterial infections in human patients should: 1) be highly specific to and inhibit a molecular pathway that is required for virulence in acute, chronic, and persistent infections; 2) be efficacious against divergent pathogens, by targeting an essential, evolutionarily conserved, and wide-spread virulence factor or pathway; 3) minimize selective resistance by not disrupting pathogen growth or viability; 4) inhibit the occurrence of AT pathogen cells; 5) be highly robust, and function at nM concentrations or less; 6) be non-toxic to and metabolically stable in host and pathogen cells; and 7) be affordably synthesized and administered. The novel compounds identified here exhibit several of these characteristics, and as such, provide a foundation for the development of next-generation anti-infective therapeutics.

Bacterial QS systems are candidate targets for the development of more effective drugs to treat acute and chronic or persistent bacterial infections, as QS pathways control the coordinated expression and production of a wide-array of virulence factors in divergent pathogens, and are dispensable for bacterial survival [11], [13]. Potential QSI therapeutics fundamentally differ from traditional antimicrobials that are bactericidal and/or bacteriostatic, as they are not expected to disrupt beneficial flora critical for health [51], nor lead to selective resistance [52]. Past approaches to identify QSI compounds against the MvfR-regulon have been predictive, focusing on structural analogues of MvfR-pathway intermediates, and of the MvfR native ligands. Analogues of anthranilic acid, the primary precursor of the HAQs and 2-AA, provided the first demonstration that pharmacological disruption of the MvfR regulon can limit *P. aeruginosa* pathogenesis [40], [53]. Other compounds were shown to reduce PQS production *in vitro*, however they lack anti-infective activity [54]–[58]. Similarly, PQS and HHQ analogues reduce a subset of MvfR regulated virulence functions, yet do not provide *in vivo* efficacy in murine infection models, block bacterial cell persistence, or show activity against multidrug-resistant *P. aeruginosa* strains [59]–[61]. In addition, *P. aeruginosa* can modify ligand-based MvfR inhibitors into MvfR activators [62], so drugs based on these compounds could ultimately increase virulence.

In contrast to these predictive approaches, our whole-cell HTS activity-based strategy is not predicated on known QS pathway molecules, and as such, should identify compounds only on their MvfR-regulon QSI activity, independent of structure. Using this strategy and chemical libraries of ∼300,000 small molecular weight compounds, we initially isolated 390 QSIs. We then used a genetic readout system combined with LC/MS to distinguish the inhibitors for robustness and specificity of MvfR pathway inhibition, which validated 17 compounds that belong to 7 divergent chemical families. None of these compounds had been shown to have anti-virulence activity previously, nor proposed as candidates for such. These are the first identified QSIs that inhibit a wide spectrum of MvfR regulon virulence functions, including the pro-acute virulence molecules, HHQ, PQS, and pyocyanin; and the pro-persistent signaling molecule, 2-AA. They are also highly potent, with culture IC_50_ values of 200–350 nM for HHQ, PQS, and pyocyanin, which is 10–1,000 times more potent than the previous analogue-based inhibitors [60], [62].

Several of the most robust 1st-generation molecules contain the structural backbone of a benzamide and benzimidazole (BB) moiety linked through a thioether bond. These inhibitors demonstrate the validity of our activity-based approach to identify unpredicted chemical structures to develop anti-infective therapeutics. We subsequently used an SAR analysis to define important BB substituents for enhanced QSI activity, including: 1) the aromatic ring, as in M56; 2) the electron-withdrawing substituent at the benzamide *para* position side-group, as in M34 and M59, especially for pyocyanin production; and 3) the increased bulk of the *para* substituent on the benzamide moiety of a nitrobenzimidazole derivative, from chlorine to cyano to bromo to iodo, with the M64 phenoxy derivative having the highest activity of all compounds tested. This highly robust 2^nd^-generation inhibitor notably has significant therapeutic efficacy against both acute and persistent infections in mice, with or without combinatorial antibiotic therapy. In addition, M64 is the first compound identified to inhibit MvfR regulation in divergent *P. aeruginosa* isolates, including currently untreatable multidrug or pan-resistant strains. Note, this suggests that M64 uptake or action does not appear to be limited in MDR clinical strains that potentially exhibit efflux or influx pumps modifications. Additionally, although M64 contains a nitroaromatic residue, it is not cytotoxic to macrophages (Fig. 6).

Inhibitors of the *P. aeruginosa* LasR-regulated QS also have *in vitro* and *in vivo* anti-virulence potential [11]–[13]. Nonetheless, these reagents may have less practical import and clinical applicability than the MvfR inhibitors, as *P. aeruginosa*-human isolates often carry *lasR* mutants. Such mutants may contribute to the fitness of chronic and persistent infections [22], [23], and could cause treatment failure of LasR-specific inhibitors. These *lasR* cells are likely “cheaters” that benefit from non-mutant cooperators, and do not overcome wild type *P. aeruginosa* cells in environments lacking selective pressure for LasR activity [52]. In addition, the MvfR pathway product, 2-AA, promotes *lasR* mutant accumulation [7], so QSI compounds that target MvfR should restrict lasR cheaters from non-mutant cooperators. Moreover, combinatorial therapy of MvfR and LasR inhibitors could enhance target spectrum and clinical potential in acute infections.

Our results show that MvfR is not required for short term bacterial survival in the host, therefore it is not surprising that M64-treated animals do not clear PA14 cells faster than untreated controls in the short term. The higher PA14 clearance observed at day 11 in M64-treated versus not treated animals may suggest a potential fitness advantage for M64-resistant cells for long-term survival in the host. However, we did not observe any M64-resistant cells at day 11 from tissues of animals infected and treated with M64 (data not shown), indicating no clear fitness advantage for M64-resistant mutants *in vivo*. This is consistent with the fact that no *mvfR* mutants have been reported in infection sites. Of greater concern are wild-type persister cells, and M64 greatly restricts persister appearance and prevents tissue re-colonization after antibiotic treatment is stopped. Re-colonization may ultimately be responsible for relapsing infection even though, in the setting presented, relapsing infection was not lethal. Transposing these data to a clinical perspective, relapsing infection that occurs after antibiotic treatment arrest may increase patient morbidity/mortality, especially in the case of immunocompromised individuals, and could serve as a source of nosocomial infection as patients remain infected for longer periods of time.

M64 directly binds to MvfR in 1∶1 stoichiometry and inhibits *pqs* operon expression by reducing MvfR binding to the *pqsA* promoter, independently of PQS. We propose that M64 induces a non-productive conformational change in MvfR to interrupt effective ligand binding to the native binding domain. Elucidation of the exact M64 binding site and co-crystalization should aid in the design of enhanced anti-MvfR compounds. That M64 does not rescue the survival of macrophages or mice infected with virulence-attenuated *mvfR* mutant cells further confirms that M64 directly targets MvfR, and its anti-virulence efficacy is not an off-target effect. These results also show that pharmacological *in vivo* inhibition of MvfR function effectively reduces acute and persistent *P. aeruginosa* infections. Although M64 and the QS molecule 2-AA [7] both function as MvfR-regulon inhibitors, these two molecules act differently. There are several key differences between 2-AA and M64: 1) 2-AA promotes the accumulation of AT cells (Fig. 3b and [8]) and host tolerance to infection leading to bacterial persistence [6] whereas M64 prevents these phenotypes (Fig. 3b and 9); 2) 2-AA acts upon at least one of the PQS enzymes of the *pqsABCDE* operon [7], while M64 does not (Fig. 5a); and 3) M64 is a much more potent MvfR regulon inhibitor than 2-AA as M64 IC_50_ for the *pqsA* gene expression inhibition is ∼1,300 times lower (Fig. S7).

M64 exhibits additive effects for *P. aeruginosa* infections when combined with sub-therapeutic doses of the clinically relevant antibiotic, ciprofloxacin. M64 is also highly efficacious when used in monotherapy in burn and lung acute *P. aeruginosa* infections in mice. Chronic or persistent infections are often refractory to traditional antibiotics and/or host defense killing mechanisms due to AT and persistent cell subpopulations. Biofilms could form a protective environment for this subpopulation of cells, shielding them from the immune system. The MvfR regulon is reported to control biofilm formation, however in this study the contribution of biofilm or the efficacy of M64 against biofilm formation in our *in vivo* studies was not assessed. Although many studies have focused on targeting biofilms specifically [63], [64], only very few focused on specifically targeting persister/AT cells. Moreover, current anti-infectives neither target nor limit such cells, leaving a reservoir for re-initiation of infection that underlies chronic/persistent and relapsing infections. AT cells are clinically important, as antibiotics often fail to clear pervasive infections, and the contribution of tolerance to treatment failure and mortality can be as significant as antibiotic resistance. As such, there is considerable need to identify anti-AT compounds that: 1) prevent the formation of AT cells; 2) allow them to be killed; or 3) prevent them from “waking up” when an antibiotic is removed, which can require long-term continuous administration of the anti-infective to assure full clearance. This is both financially costly, and potentially deleterious to the host's natural microbiome. Strategies for killing AT cells include augmentation of antibiotic uptake with sugars [65], stimulation of reactive oxygen species production [66], activation of endogenous proteases [67], or waking-up AT cells with small molecules [68]. Here, M64 prevents AT formation, and in combination with ciprofloxacin, eliminates bacterial rebound and promotes full bacterial clearance. As such, M64 and related compounds provide for the development of robust anti-virulence therapeutics to treat acute, chronic, persistent, and pervasive relapsing infections, in combination with sub-therapeutic levels of traditional antibiotics. In addition, that M64 is efficacious in monotherapy and interferes with AT cell formation, suggests that prophylactic application of M64-based derivatives could reduce antibiotic use. Although M64 will not enter clinical trials directly, it will provide a basis for the development of next generation anti-virulence compounds that contain a similar core structure. As such, it will guide medicinal chemistry efforts to improve solubility and eliminate the potentially problematic nitro group to enhance drug characteristics and ultimately become a new clinical weapon against acute and persistent bacterial infections.

In conclusion, our MvfR-regulon QSI compounds are candidates for the development of next-generation anti-infective/anti-virulence therapeutics, as: 1) they inhibit expression of the MvfR virulence regulon; 2) they do not alter bacterial cell viability or growth; 3) they inhibit the pathogenicity of MDR clinical isolates; 4) they reduce *P. aeruginosa* virulence in clinically relevant mouse infection models, and compound with antibiotics to block persistent infections; and 5) they restrict the occurrence of AT bacterial cells that underlie chronic and persistent host infections. M64, a 2^nd^ generation inhibitor identified by SAR, is the first identified compound that exhibits significant *in vivo* therapeutic efficacy against both acute and persistent mammalian infections. Furthermore, that LTTRs regulate virulence regulons in divergent bacterial pathogens [69], and QS functions throughout the eubacteria and archaebacteria, suggest that M64-based anti-infectives could have broad clinical potential against a wide-range of bacterial pathogens.

## Materials and Methods

### Ethics statement

Animal procedures were performed according to the animal protocols, 2006N000093/2 and 2005N000387/6, approved by the Massachusetts General Hospital Institutional Animal Care and Use Committee. The two protocols conform to the USDA Animal Welfare Act, PHS Policy on Humane Care and Use of Laboratory Animals, the “ILAR Guide for the Care and Use of Laboratory Animals” and other applicable laws and regulations.

### Bacterial strains, growth conditions, and gene constructs

UCBPP-PA14 (PA14) is a Rif^R^ *P. aeruginosa* human clinical isolate [20]. All mutant strains including *mvfR* [19] are isogenic to UCBPP-PA14. Unless noted, bacteria were grown at 37°C in LB broth or on LB agar plates containing 75 µg/mL tetracycline, 100 µg/mL rifampicin, and 300 µg/mL carbenicillin,.

*mvfR*-pPqsABCD bacteria, which have constitutive and MvfR-independent *pqs* operon expression, were generated by cloning the *pqsABCD* operon into pDN18 and electroporating this construct into *mvfR* cells. These bacteria were grown for 6 h in 100 µM of experimental compound, or in 0.2% DMSO as control.

The *P. aeruginosa* clinical isolates, SH2, Shr12, Shr22, Shr23, Shr33, Shr34, Shr37, Shr40, and Shr42, were obtained from Shriners Hospital, Boston MA.

P*pqsA*-GFPASV was previously described [38].

The growth kinetics of PA14 WT or PA14 P*pqsA*-GFP cells were recorded using an automated 96-well plate reader (Sunrise Tecan, Switzerland) at 37°C with 10 s of circular shaking every 15 min, followed by 10 s of settling at which time OD_600 nm_ was detected.

The *pqsA-sacB* reporter gene was generated by fusing the *pqsA* promoter to the *Bacillus subtilis sacB* gene [70]. The *pqsA* fragment was amplified using PA14 chromosomal DNA and primer pairs 5′GACTAGTCGAGCAAGGGTTGTAACGGTTTTTG3′ and 5′GAAGATCTGACAGAACGTTCCCTCTTCAGCGA3′. The *sacB* fragment was amplified using pKOBEG-sacB [70] DNA and primer pairs 5′GAAGATCTATGAACATCAAAAAGTTTGCA3′ and 5′AAACTGCAGGTTGATAAGAAATAAAAGAAAATGCC3′. The PpqsA and sacB fragments were digested with SpeI/BglII and BglII/PstI, respectively, and ligated to SpeI/BglII-digested pCTX (TetR). The resultant construct was eletroporated into *E. coli* SM10 lambda pir and CTX-PpqsA-sacB was integrated into the PA14 chromosome [71]. PA14:CTX-P*pqsA*-sacB clones were selected on Rif/TetR plates and confirmed by PCR.

### HTS

That ligand-bound MvfR binds to and activates the *pqsA* promoter [16] provided the basis for a biological reporter assay for a high throughput screen (HTS), using PA14 cells carrying a *pqsA-sacB* reporter gene. *pqsA* encodes an anthranilate-coenzyme A ligase that activates anthranilic acid and catalyzes the first committed step to HAQ production [72], [73], and is positively regulated by the MvfR protein. *sacB* encodes levansucrase, which causes toxicity when cells are grown in sucrose, and has been incorporated into allelic exchange vectors to provide counter-selection [74]. Here, the PA14:*pqsA-sacB* cells die when MvfR activates the *pqsA* promoter, so compounds that suppress *pqsA* expression allow growth on sucrose. MvfR inhibition results in reduced *sacB* expression and viable growth, as determined by absorbance.

Using a plate reader and OD_600 nm_ as readout, the *pqsA-sacB* construct proved successful in a pilot high-throughput experiment using 4-CABA, an AA analog that effectively inhibits the MvfR regulon [40], as a positive control.

Overnight PA14 *pqsA-sacB* cultures were subcultured and grown to mid-logarithmic phase, centrifuged, washed, resuspended to a final OD_600 nm_ of 0.05 in LB minus NaCl and plus 10% sucrose, and 30 µl of cells were aliquoted into 384-well plates using a Matrix *Well*Mate. 1.5 mM 4-CABA, a previously described PqsA inhibitor [40], was added to one plate column as the positive control, with another column left compound free for the negative control. 300 nl of a library compound in DMSO was added to each plate via an Epson compound transfer robot, to give a final well concentration of 50 µg/ml. Each library plate was screened in duplicate. After 8 h incubation at 37°C, the OD_600 nm_ was determined for each well using an EnVision plate reader (Perkin-Elmer). The relative inhibition of each compound was from its z-score [75]. This analysis normalizes candidate inhibitory compounds on a plate to plate basis, and corresponds to the standard deviation from the mean plate value.

284,256 compounds in libraries at the Institute of Chemistry and Cell Biology (ICCB)-Longwood screening facility (Fig. S1) were screened in duplicate to identify 532 potential robust inhibitors with strong z-scores. Of these, 390 had limited potential liability, based on their structures. These compounds were tested in a secondary screen at ∼50 µg/mL and ∼25 µg/mL using a reporter construct of the *pqsA* promoter fused to a short half-life GFP gene [38] as described [7], such that quantitative quenching of fluorescence corresponded to *pqsA* promoter repression. MvfR inhibition results in reduced GFP expression. This assay eliminated potential false positives, including compounds that negatively affect SacB activity (Fig. S2). Each compound was concomitantly assessed for growth (OD_600 nm_).

### HAQs and pyocyanin quantification

HAQs and 2-AA were quantified in bacterial culture supernatants by LC/MS [16], [76]. Pyocyanin levels were quantified by measuring OD_520 nm_ of chloroform-extracted cultures [77].

### Persister cell assay

*P. aeruginosa* PA14 or *mvfR* mutant cells were grown with shaking and aeration to mid-logarithmic phase in LB broth, minus and plus exogenous compound. Before antibiotic addition, and as the normalization reference, a culture aliquot was diluted 10^6^ fold in fresh LB (pre-antibiotic) and plated on LB agar for CFU quantification. The remainder of the culture was treated with meropenem to a final concentration of 100×MIC (Minimum Inhibitory Concentration; 10 mg/L) or 5 mg/L amikacin, 0.1 mg/L levofloxacin, or 0.4 mg/L ciprofloxacin. At 16 h post-antibiotic, culture aliquots were washed 2 times in fresh LB to remove antibiotic carry-over, 10-fold serially diluted in LB broth, and plated on LB agar for CFU quantification. This procedure was repeated at 24 h post-AB to ensure that a killing plateau was reached. The persisters fraction was determined as the ratio of normalizers (pre-antibiotic) divided by persisters (24 h post-antibiotic).

### Recombinant MvfRc91 purification

The *mvfR* gene from residue 91 at nucleotides 271 to 273, to the MvfR stop codon at nucleotide 999, plus 129 bp downstream, was cloned into the *Nde1* and *Xho1* sites of pET16B, to generate pET16B-MvfRc91. *E. coli* BL21(DE3) cells harboring pET16b-MvfRc91 were grown at 37°C to OD_600 nm_ 0.6 [16]. His-tagged MvfRc91 expression was induced with 0.5 mM IPTG at 20°C for 16 h, and the cells were harvested by centrifugation. The bacterial pellet was resuspended in Tris buffer (20 mM Tris-HCl, 300 mM NaCl, pH8.0) with 10 mM imidazole, and lysed by sonication. The soluble fraction was collected by centrifugation and filtration, and separated on a Ni-NTA column equilibrated with Tris buffer containing 50 mM imidazole. After column wash, the His-tagged MvfRc91 protein was eluted with a 0.1 M–1.0 M imidazole gradient. The MvfRc91fractions were pooled and dialyzed in phosphate buffer (pH 8.0) with 300 mM NaCl and 2.5 mM β-mercaptoethanol.

### Chromatin immunoprecipitation (ChIP)

The ChIP assay was performed as described in [78] using VSV-G-tagged MvfR in PA14 cells. To construct the MvfR - VSV-G integration vector pP30ΔFRT-MvfR – VSV-G, the 316 bp fragment corresponding to *mvfR* 630–945 region was amplified by PCR using forward primer *mvfR* HindIII (5′-GACGTAAGCTTGGTCAGCGACAAGGTGCTCTTC-3′) and reverse primer *mvfR*_NotI (5′-GAAATGCGGCCGCCTGCACCGTTTCGACGATGCTCGG-3′). The PCR product was then treated with HindIII and NotI and cloned into the HindIII and NotI sites of pP30ΔFRT [79]. PA14 expressing MvfR-VSV-G was obtained via conjugation of PA14 with *E. coli* S17-1 λpir carrying the pP30ΔFRT-MvfR - VSV-G plasmid. Conjugants with chromosomally-integrated plasmid were selected on LB plate containing 30 µg/ml gentamicin. Plasmid backbone excision was performed by transforming plasmid pFLP2, which encodes FLP recombinase. Expression of the MvfR – VSV-G protein was confirmed by western blot analysis using a rabbit anti – VSV-G primary antibody (Sigma–Aldrich) and a goat anti-rabbit HRP-conjugated secondary antibody (GE Healthcare). Loading control was performed for housekeeping protein RpoD detected with a mouse anti-RpoD primary antibody (Neoclone) and a sheep anti-mouse HRP-conjugated secondary antibody (GE Healthcare Science).

Binding of tagged MvfR protein to the non-MvfR-regulated *rpoD* promoter DNA was used as the negative control. 5 ml culture aliquots were inoculated at OD_600 nm_ 0.03, and grown at 37°C to OD_600 nm_ 0.75, minus and plus 0.24 µM M64, and minus and plus 38 µM PQS. Cross-linking and ChIP were as described [78]. Quantitative PCR used oligonucleotides to the *pqs* operon, and to the *rpoD* promoter as negative control [31], [80]. MvfR binding was expressed as the percent of total input DNA. Data were averaged from at least 3 replicates.

### Isothermal titration calorimetry (ITC)

ITC experiments were performed using a VP-ITC (Microcal). Ligand and protein were in 100 mM phosphate buffer (pH 8.0) plus 2.5 mM β-mercaptoethanol and 10% methanol. For individual titrations, 10 µl of 200 µM M64 was injected using a computer-controlled microsyringe at 180 second intervals into 1.5 ml of 19 µM MvfRc91, with the sample cell stirred at 300 rpm, and the heat produced was measured at 25°C. The heat originating from M64 injection into buffer alone was subtracted from the raw data. The dissociation constant was calculated using Origin Software (Microcal) by plotting heat per injection (µJ) versus the titrated MvfRc91/M64 stoichiometry.

### Macrophage cytotoxicity

PA14 or *mvfR* bacterial cultures were grown to OD_600 nm_ 2.0, minus and plus 50 µM experimental compound. Raw264.7 macrophage cells were cultured in Dulbecco's modified Eagles medium (DMEM) containing 10% FCS, 2 mM glutamine, and antibiotic-antimycotic; washed with PBS (Mg^2+^ and Ca^2+^ free); resuspended in antibiotic-free medium; and infected with 100 MOI of bacteria. Following 3 h incubation at 37°C under 5% CO_2_, macrophages were washed and incubated for a further 3 h with DMEM containing polymixin B and gentamycin to kill extracellular bacteria. Raw264.7 viability was assessed using the MTT (3-[4, 5-dimethyl-2-thiazolyl]-2, 5-diphenyl-2*H*-tetrazolium bromide) assay for early detection of eukaryotic cell death [81]. Briefly, macrophages were incubated in 200 µl PBS containing 200 µg/ml MTT (Sigma-Aldrich) in a 96-well culture plate for 2 h at 37°C under 5% CO_2_, and the dissolved MTT was converted to insoluble purple formazan via intracellular mitochondrial activity. The supernatant was removed and the cells were lysed for 10 min with 95% isopropanol and 5% formic acid. Converted dye absorbance was measured at OD_570 nm_, with OD_690 nm_ as the reference wavelength, in a Sunrise plate reader (Tecan Group Ltd, Männedorf, Switzerland). Per cent infected cell viability was calculated by dividing the OD_570 nm_ of infected versus uninfected culture.

### Murine burn and infection model to address anti-virulence and anti-persistence compound efficacy

A murine thermal injury model was used to assess bacterial pathogenicity in 6–7 wk-old CD-1 mice, as described [20]. Briefly, animals were anesthetized with Xylazine (13 mg/kg, i.p.) and Ketamine (87 mg/kg, i.p.), thermally injured (5–8% of body surface) on the shaved abdomen dermis, and intra-dermally infected into the burn eschar.

To assess MvfR inhibitory compound activity in acute infection, animals were inoculated with 5×10^4^ PA14 cells in 100 µl of 10 mM MgSO_4_; and injected IV into the tail vein with M64 (4 mg/kg in 15% cremophore) and/or ciprofloxacin (10 mg/kg or 0.4 mg/kg), twice a day for up to 6 or 4 days post-infection, respectively, for M64 or ciprofloxacin. Mice survival was assessed over the course of 7 or 14 days, with 10 animals per experimental group, and CFUs in adjacent or underlying muscle were quantified at 2, 3, 4, 6, 8, 11, and 14 days post-infection, as described [7]. Samples from underlying muscle represent assessment of bacterial CFUs at the site of inoculation, whereas the adjacent muscle samples provide a read out of the bacterial dissemination from the site of inoculation. A sub-therapeutic concentration of ciprofloxacin (0.4 mg/kg) was used to assess M64 and ciprofloxacin additive effect. Kaplan-Meier statistical analysis was performed using Prism Graphpad software.

To assess MvfR-regulon inhibitor efficacy in persistent infections, persistent nonlethal infections were produced in burned mice by using a lower bacterial inoculum than the one used above. Mice were inoculated with ∼6×10^3^ PA14 cells in 100 µl of 10 mM MgSO_4_. Ciprofloxacin (10 mg/kg) plus or minus, M64 (4 mg/kg in 15% cremophore) were IV injected into the tail vein twice a day for up to 6 or 4 days post-infection for M64 or ciprofloxacin, respectively. CFUs in adjacent or underlying muscle were quantified at 2, 3, 4, 6, 8, 11, and 14 days post-infection, as described [7]. MIC was calculated via the multivariate E-Test (Biomerieux) [7].

### Mouse lung infection model

M64 *in vivo* efficacy was further assessed in a murine lung acute infection model [82]. 6 wk-old CD-1 mice were anaesthetized with Xylazine (13 mg/kg, IP) and Ketamine (87 mg/kg, IP), and intranasally inoculated with 20 µL of 5×10^6^ PA14 or 8×10^6^ *mvfR* mutant cells. M64 (4 mg/kg or 12 mg/kg) was injected IV at 2, 4, 8, and 12 h, followed by injections twice daily through 4 days post-infection. Animals were held in a vertical position for 3–5 min to facilitate distal alveolar migration of the bacteria by gravity. Mice survival was assessed over 6 days, with 10 or more animals per experimental group. Kaplan-Meier statistical analysis was performed using Prism Graphpad software.

### *In vivo* molecular MR Imaging to address anti-virulence compound efficacy

*In vivo* molecular magnetic resonance imaging positive contrast method exploits the chemical shift induced by ultra-small super-paramagnetic iron oxide (USPIO) nanoparticles, known generically as Ferumoxtran-10 commercially and as Combidex in the U.S. (Advanced Magnetics, Cambridge, MA). We used the USPIO nanoparticles as the molecular imaging MRI contrast agent. Six weeks old CD-1 mice were anesthetized with Xylazine (13 mg/kg, IP) and Ketamine (87 mg/kg, IP) and a leg thermal injury of ∼8% total burn surface area was produced on the right thigh muscle. Six hours post-burn and infection 500 mg of Ferumextron-10 suspension was injected by intravenous injection in the tail vein. Mice were randomized into one experimental and one control group (N = 6 per group). The experimental group consisted of burned and infected mice, injected with USPIO and injected with the anti-infective compound M64. The control group consisted of burned and infected mice injected with USPIO. The mice were imaged 12 hour post-burn and infection. We imaged the accumulation of USPIO-labeled macrophages at the *P. aeruginosa* infection site in the mouse burn and infection model. Briefly, imaging was performed in a 4.7 T horizontal magnet (20 cm bore, Bruker Avance console) equipped with a 39 G/cm gradient system, using a custom volume coil of 3 cm inner diameter and 10 cm active length. This set-up permits high B1 for extended periods of time necessary for T_2ρ_ and provides extended homogeneity. Imaging was performed with RARE acceleration factor two. The ORI-T_2ρ_ sequence used a spin-locking pulse block for relaxation in the rotating frame between the 90° and 180° RF pulses of the RARE sequence. Magnetization inversion was achieved with 180° adiabatic full passage pulses using HS4 adiabatic pulses (3 ms pulse duration, BW = 7 kHz) [83]. The spin-locking block was implemented with the MLEV-4 scheme (12 ms duration of spin-lock) [84]. Water and fat were suppressed using frequency-selective ten-lobed sinc pulses (400 Hz pulse bandwidth for water, 800 Hz for fat), followed by spoiling gradients to dephase the transverse magnetization. Typical parameters were RARE acceleration factor 2, effective echo time (TE) 9.93 ms, repetition time TR 2240 ms, with 8 averages. Anatomical reference images were acquired with RARE or proton-density weighted FLASH (fast-low angle shot) imaging. Negative contrast was achieved with a series of FLASH images with increasing echo time for T_2_* weighting, with typical values α = 35°, TR = 500 ms, TE = 4, 6, 8, 12, and 14 ms. The same slice prescription was used for all sequences. Typically, 10 axial slices were acquired in the burned region (1 mm thickness, 1.5 mm gap, 3×3 cm FOV, 128×128 matrix size, 8 averages). Typical MR imaging times were 1.3 hr per animal.

### MIC determination and antibiotic sensitivity/resistance profiling

PA14 MIC for antibiotic treatment was as described [85]. Antibiotic sensitivity/resistance profiles were determined using the disc diffusion method [86].

### Statistical analysis

Data from 3 or more independent experiments were analyzed using the Student's t-test or one way ANOVA with Dunnett's post-test when required, and animal data were analyzed using the Kaplan-Meier survivability test. P values<0.05, <0.01, and <0.001 were considered statistically significant, very significant, and highly significant, respectively.

## Supporting Information

Figure S1 **Experimental strategy using whole cell High Throughput Screening (HTS) and functional assays to identify MvfR regulon inhibitory compounds.**
(JPG)

Figure S2 **HTS identified MvfR-regulon inhibitors quench fluorescence of pqsA-GFP expression without impacting bacterial growth.** **a.** Cells fluoresce when the *pqsA*-GFP reporter gene is activated via MvfR. Fluorescence was unaltered in response to HTS inhibitors at 50 µg/ml, versus the 0.2% DMSO positive control (black line). Similar results to those from LB medium were obtained with the low autofluorescence medium TSB (*data not shown*). **b.** Growth, measured by OD_600 nm_, was unaltered by each of these compounds. Note that these compounds represent those with strong z-scores in the initial HTS.
(JPG)

Figure S3 **M64 inhibitory efficacy on HHQ, PQS, and pyocyanin production in** ***P. aeruginosa***. HHQ, PQS, and pyocyanin production were determined in response to increasing concentrations of M64 (5 nM to 100 µM). Data represent the average of at least two replicates.
(JPG)

Figure S4 **Inhibitors do not affect** ***P. aeruginosa*** **growth even at later growth stage.** PA14 growth curves in presence of two representative inhibitors, M64 and M31. Growth curves of PA14 + 0.04% DMSO (black), PA14 + 20 µM M64 (red) and PA14 + 20 µM M31. Data represent the average +/− SEM of three replicates.
(JPG)

Figure S5 **M64 and/or PQS do not affect MvfR levels.** Equal quantities of cells producing MvfR – VSV-G, grown in the absence (−) or presence (+) of PQS and/or M64, were probed for western blotting with antibodies specific for VSV-G epitope (upper panel) and RpoD (loading control, lower panel).
(JPG)

Figure S6 **Proposed biosynthetic pathway of HAQ, 2-AA and DHQ.** AA: anthranilic acid; 2-ABA: 2-aminobenzoylacetic acid; 2-AA: 2-aminoacetophenone; DHQ: 2,4-dihydroxyquinoline; PQS: 3,4-dihydroxy-2-heptylquinoline: HHQ: 4-hydroxy-2-heptylquinoline. The biosynthetic pathway of HAQ, 2-AA and DHO is adapted from Dulcey *et al.* (2013).
(JPG)

Figure S7 **M64 and 2-AA inhibitory efficacy of pqsA expression in** ***P. aeruginosa***. *pqsA*-GFP expression was determined in response to increasing concentrations of M64 (10 nM to 100 µM) or 2-AA (180 µM to 1.5 mM). Data represent the average of at least two replicates.
(JPG)

Table S1 **M64 does not affect PA14 MIC for common clinical antibiotics.** PA14 cultures plus or minus 2 mM M64 were incubated for 24 h in meropenem, carbenicillin, kanamycin, ciprofloxacin, amikacin, imipenem or levofloxacin; and scored for MIC.
(JPG)

Table S2 **Bacterial strains used in this study.**
(JPG)
