# Supplementary figures and images for: Identification of Anti-virulence Compounds That Disrupt Quorum-Sensing Regulated Acute and Persistent Pathogenicity
Source: PLoS Pathog. 2014 Aug 21;10(8):e1004321. doi: 10.1371/journal.ppat.1004321 (PMC4140854; doi:10.1371/journal.ppat.1004321)

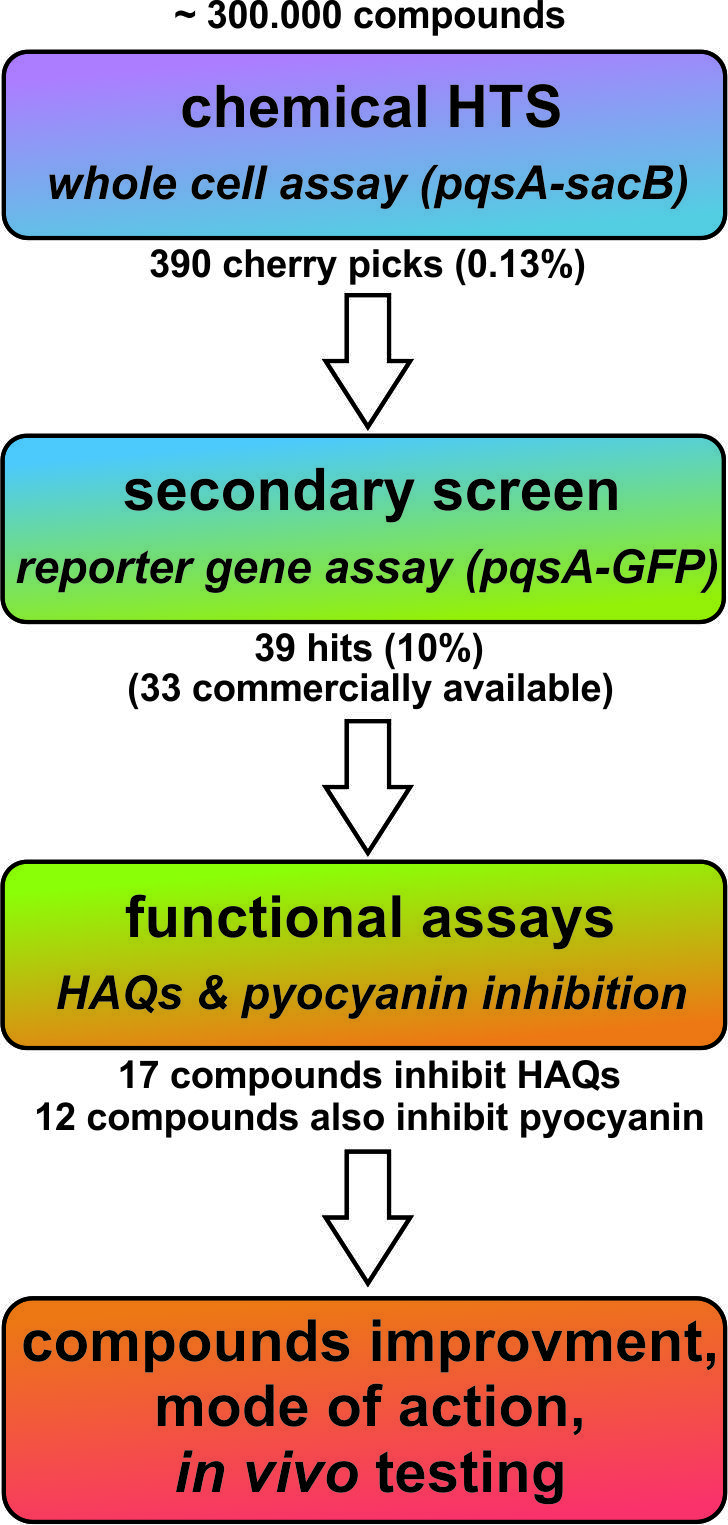

Supplement: Figure S1 — Experimental strategy using whole cell High Throughput Screening (HTS) and functional assays to identify MvfR regulon inhibitory compounds. (JPG) [file ppat.1004321.s001.jpg]

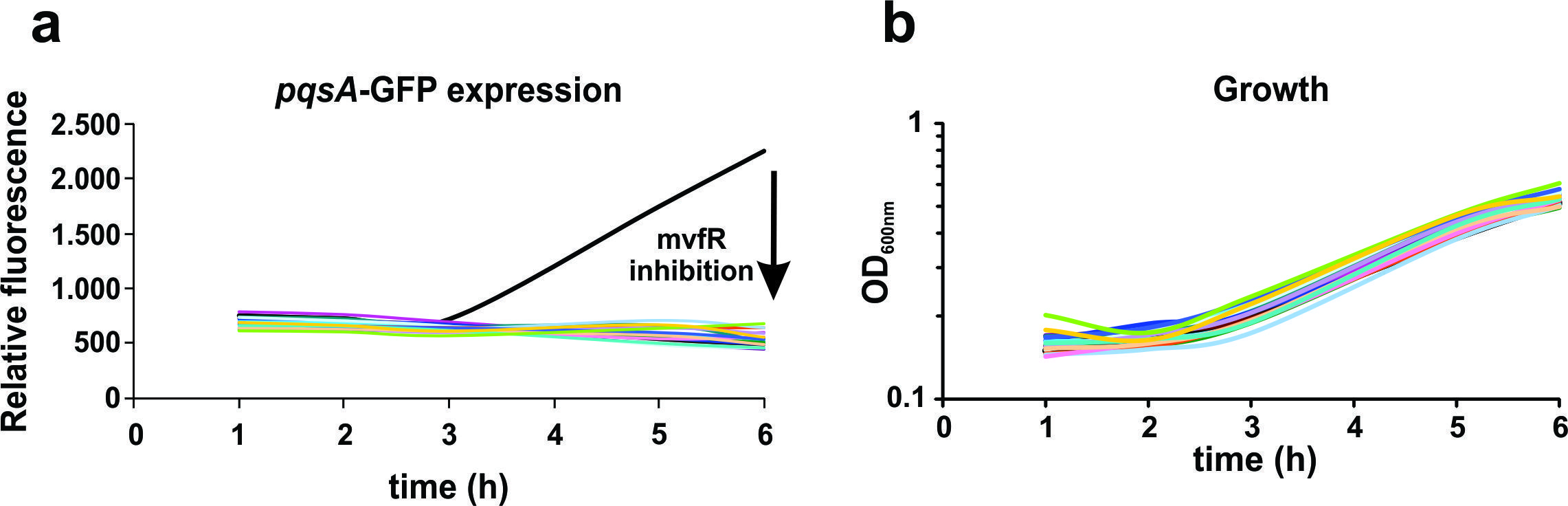

Supplement: Figure S2 — HTS identified MvfR-regulon inhibitors quench fluorescence of pqsA-GFP expression without impacting bacterial growth. a. Cells fluoresce when the pqsA-GFP reporter gene is activated via MvfR. Fluorescence was unaltered in response to HTS inhibitors at 50 µg/ml, versus the 0.2% DMSO positive control (black line). Similar results to those from LB medium were obtained with the low autofluorescence medium TSB (data not shown). b. Growth, measured by OD600 nm, was unaltered by each of these compounds. Note that these compounds represent those with strong z-scores in the initial HTS. (JPG) [file ppat.1004321.s002.jpg]

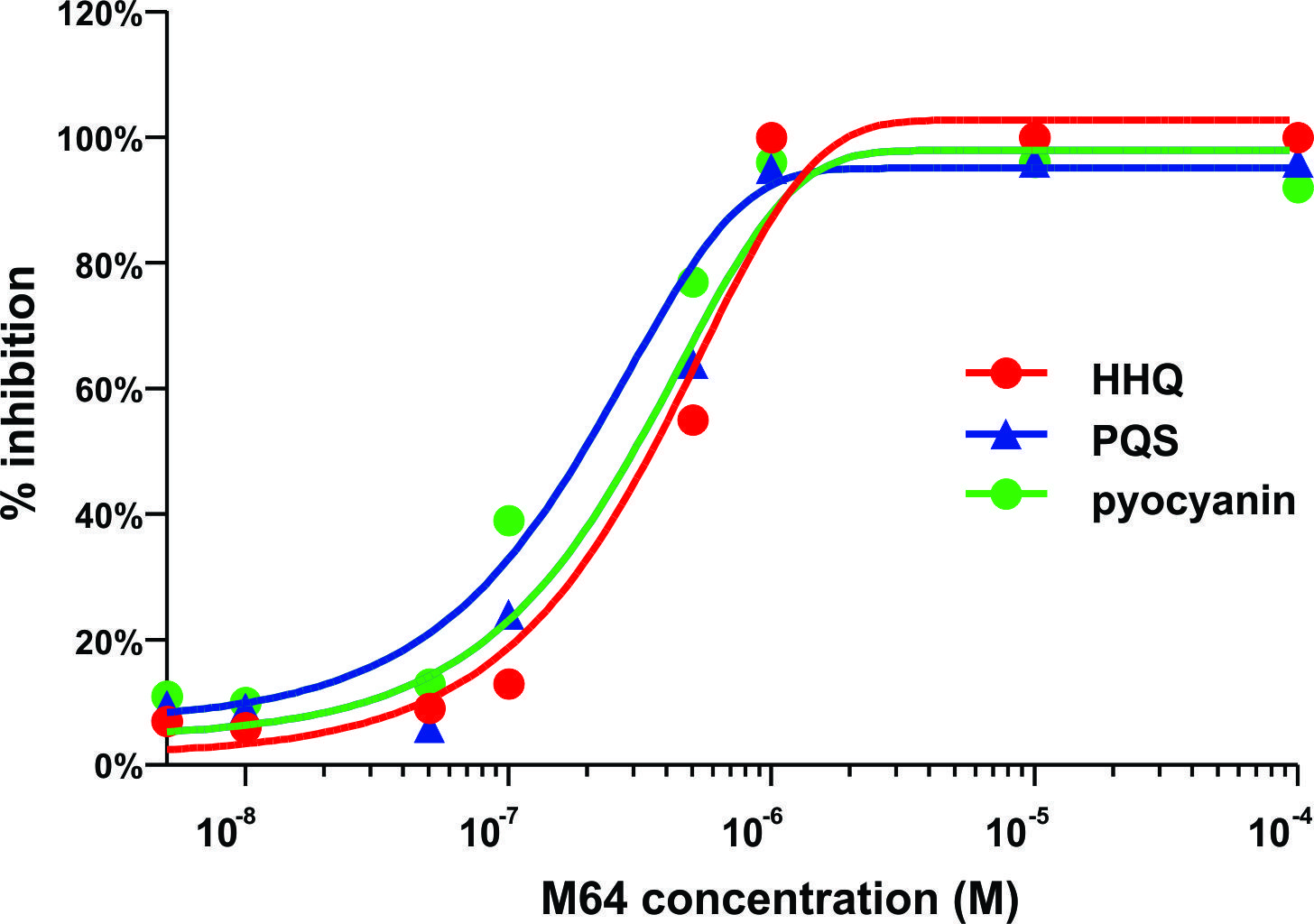

Supplement: Figure S3 — M64 inhibitory efficacy on HHQ, PQS, and pyocyanin production in P. aeruginosa. HHQ, PQS, and pyocyanin production were determined in response to increasing concentrations of M64 (5 nM to 100 µM). Data represent the average of at least two replicates. (JPG) [file ppat.1004321.s003.jpg]

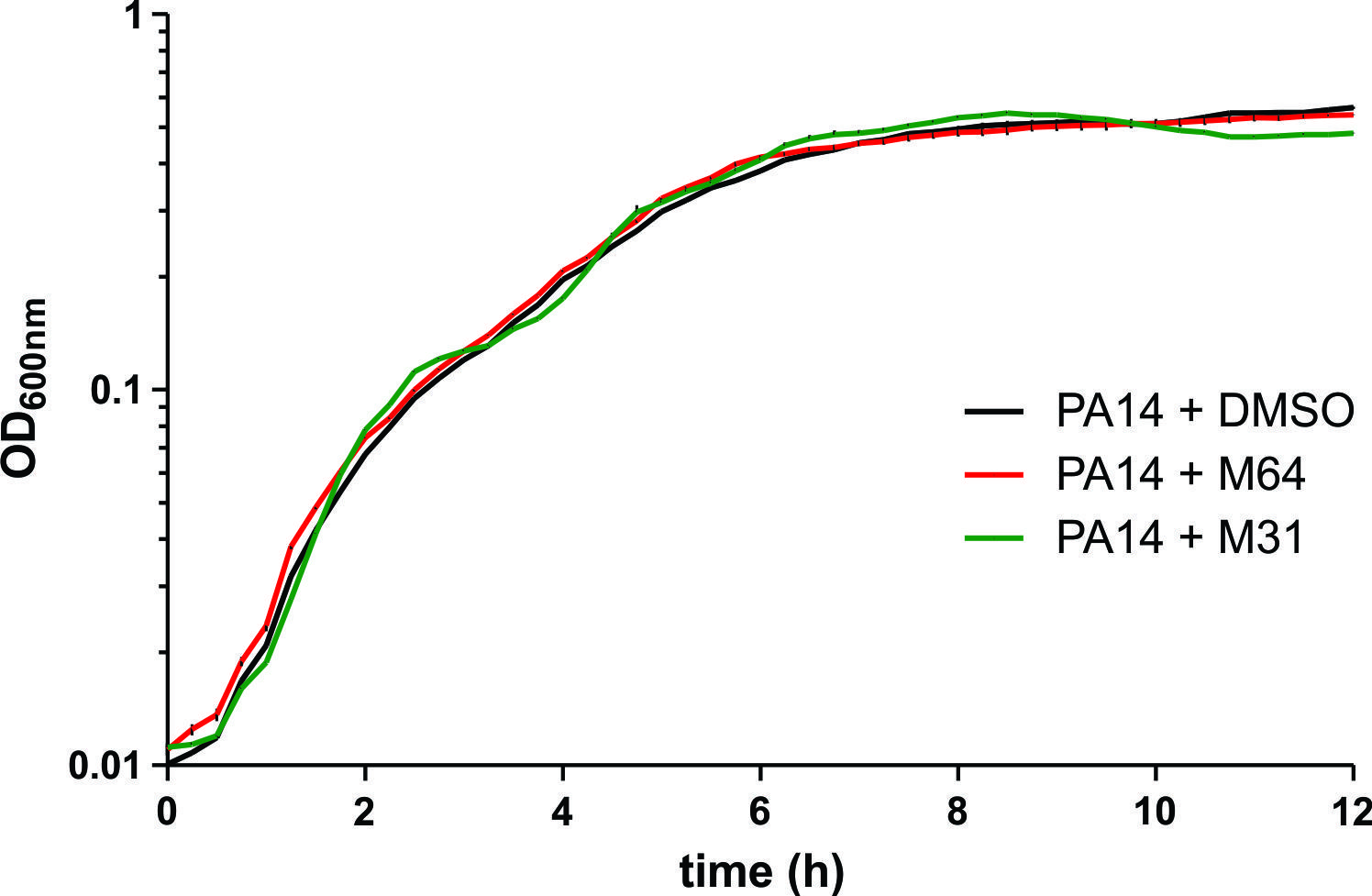

Supplement: Figure S4 — Inhibitors do not affect P. aeruginosa growth even at later growth stage. PA14 growth curves in presence of two representative inhibitors, M64 and M31. Growth curves of PA14 + 0.04% DMSO (black), PA14 + 20 µM M64 (red) and PA14 + 20 µM M31. Data represent the average +/− SEM of three replicates. (JPG) [file ppat.1004321.s004.jpg]

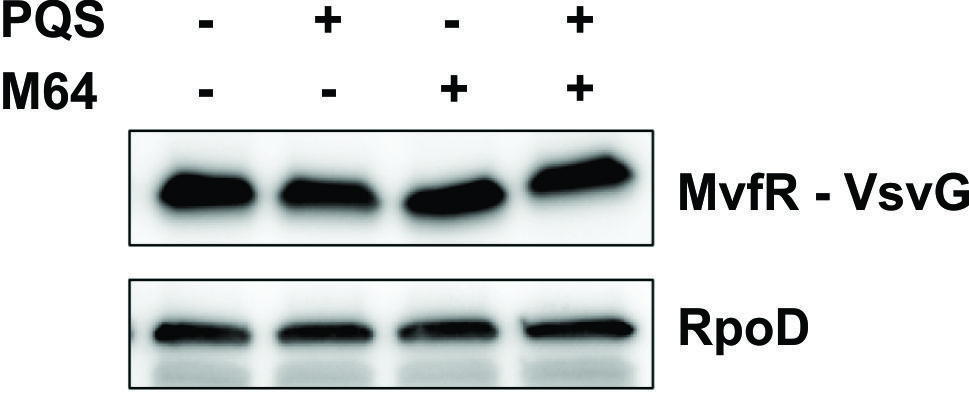

Supplement: Figure S5 — M64 and/or PQS do not affect MvfR levels. Equal quantities of cells producing MvfR – VSV-G, grown in the absence (−) or presence (+) of PQS and/or M64, were probed for western blotting with antibodies specific for VSV-G epitope (upper panel) and RpoD (loading control, lower panel). (JPG) [file ppat.1004321.s005.jpg]

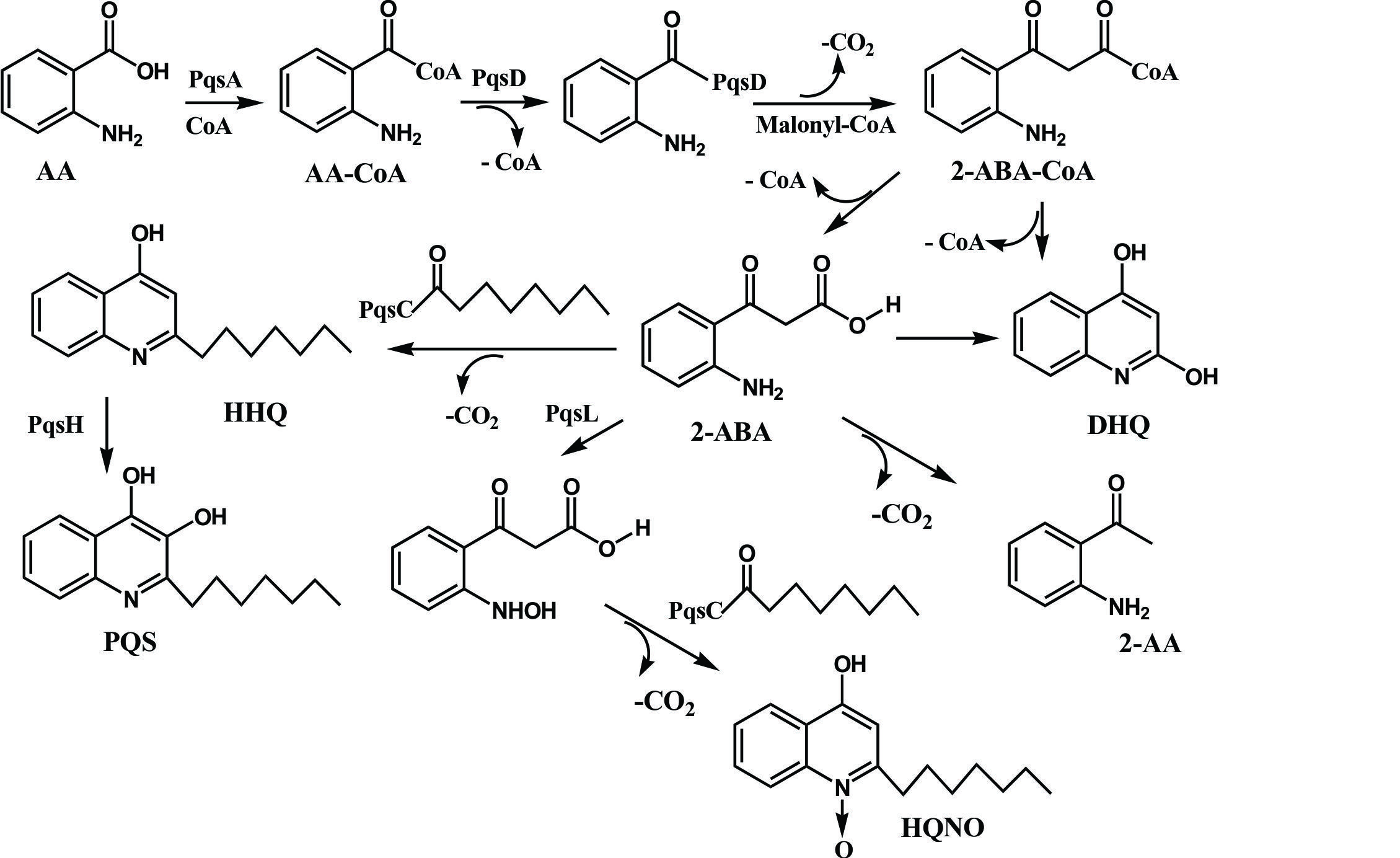

Supplement: Figure S6 — Proposed biosynthetic pathway of HAQ, 2-AA and DHQ. AA: anthranilic acid; 2-ABA: 2-aminobenzoylacetic acid; 2-AA: 2-aminoacetophenone; DHQ: 2,4-dihydroxyquinoline; PQS: 3,4-dihydroxy-2-heptylquinoline: HHQ: 4-hydroxy-2-heptylquinoline. The biosynthetic pathway of HAQ, 2-AA and DHO is adapted from Dulcey et al. (2013). (JPG) [file ppat.1004321.s006.jpg]

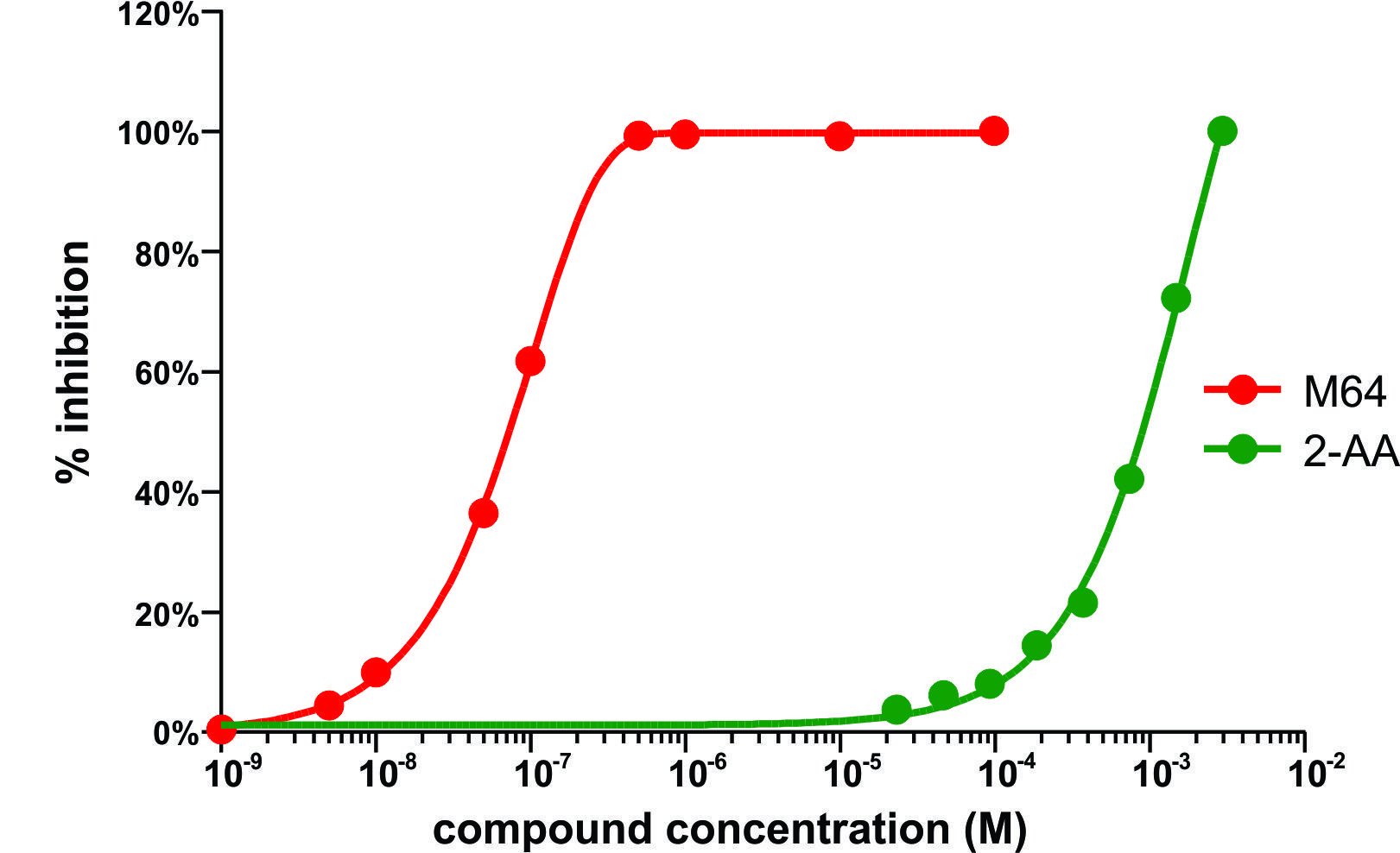

Supplement: Figure S7 — M64 and 2-AA inhibitory efficacy of pqsA expression in P. aeruginosa. pqsA-GFP expression was determined in response to increasing concentrations of M64 (10 nM to 100 µM) or 2-AA (180 µM to 1.5 mM). Data represent the average of at least two replicates. (JPG) [file ppat.1004321.s007.jpg]

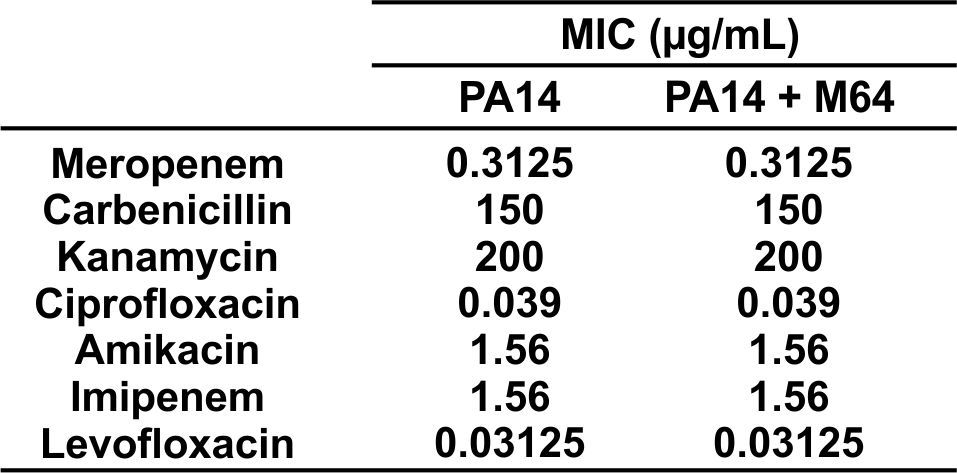

Supplement: Table S1 — M64 does not affect PA14 MIC for common clinical antibiotics. PA14 cultures plus or minus 2 mM M64 were incubated for 24 h in meropenem, carbenicillin, kanamycin, ciprofloxacin, amikacin, imipenem or levofloxacin; and scored for MIC. (JPG) [file ppat.1004321.s008.jpg]

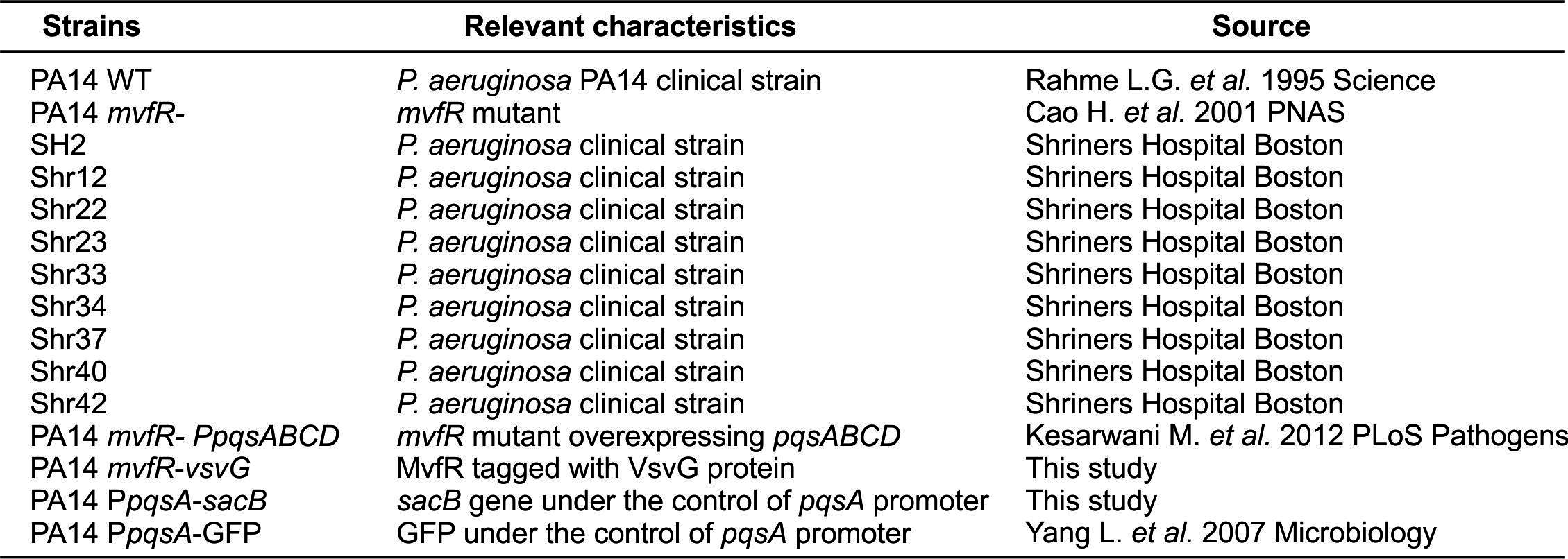

Supplement: Table S2 — Bacterial strains used in this study. (JPG) [file ppat.1004321.s009.jpg]
